# Single-cell analyses reveal evolution mimicry during the specification of breast cancer subtype

**DOI:** 10.7150/thno.96163

**Published:** 2024-05-19

**Authors:** Zhi-Jie Gao, Huan Fang, Si Sun, Si-Qing Liu, Zhou Fang, Zhou Liu, Bei Li, Ping Wang, Sheng-Rong Sun, Xiang-Yu Meng, Qi Wu, Ce-Shi Chen

**Affiliations:** 1 Department of Breast and Thyroid Surgery, Renmin Hospital of Wuhan University, Wuhan, Hubei, China.; 2 Kunming Institute of Zoology, Chinese Academy of Sciences. Kunming, Yunnan, China.; 3 Kunming College of Life Sciences, University of Chinese Academy of Sciences, Kunming, Yunnan, China.; 4 Department of Clinical Laboratory, Renmin Hospital of Wuhan University, Wuhan, Hubei, China.; 5 Breast Tumor Center, Sun Yat-Sen Memorial Hospital, Sun Yat-Sen University, Guangzhou, China.; 6 Department of Pathology, Renmin Hospital of Wuhan University, Wuhan, Hubei. China.; 7 Medical College, Anhui University of Science and Technology, Huainan, AnHui. China.; 8 Tongji University Cancer Center, Shanghai Tenth People's Hospital, School of Medicine, Tongji University, Shanghai, China.; 9 Health Science Center, Hubei Minzu University, Enshi, Hubei, China.; 10 Academy of Biomedical Engineering, Kunming Medical University, Kunming, Yunnan, China.; 11 The Third Affiliated Hospital, Kunming Medical University, Kunming, Yunnan, China.

**Keywords:** breast cancer, single-cell RNA-seq, tumor cell-of-origin, molecular subtype, tumor microenvironment

## Abstract

**Background:** The stem or progenitor antecedents confer developmental plasticity and unique cell identities to cancer cells via genetic and epigenetic programs. A comprehensive characterization and mapping of the cell-of-origin of breast cancer using novel technologies to unveil novel subtype-specific therapeutic targets is still absent.

**Methods:** We integrated 195,144 high-quality cells from normal breast tissues and 406,501 high-quality cells from primary breast cancer samples to create a large-scale single-cell atlas of human normal and cancerous breasts. Potential heterogeneous origin of malignant cells was explored by contrasting cancer cells against reference normal epithelial cells. Multi-omics analyses and both *in vitro* and *in vivo* experiments were performed to screen and validate potential subtype-specific treatment targets. Novel biomarkers of identified immune and stromal cell subpopulations were validated by immunohistochemistry in our cohort.

**Results:** Tumor stratification based on cancer cell-of-origin patterns correlated with clinical outcomes, genomic aberrations and diverse microenvironment constitutions. We found that the luminal progenitor (LP) subtype was robustly associated with poor prognosis, genomic instability and dysfunctional immune microenvironment. However, the LP subtype patients were sensitive to neoadjuvant chemotherapy (NAC), PARP inhibitors (PARPi) and immunotherapy. The LP subtype-specific target PLK1 was investigated by both *in vitro* and *in vivo* experiments. Besides, large-scale single-cell profiling of breast cancer inspired us to identify a range of clinically relevant immune and stromal cell subpopulations, including subsets of innate lymphoid cells (ILCs), macrophages and endothelial cells.

**Conclusion:** The present single-cell study revealed the cellular repertoire and cell-of-origin patterns of breast cancer. Combining single-cell and bulk transcriptome data, we elucidated the evolution mimicry from normal to malignant subtypes and expounded the LP subtype with vital clinical implications. Novel immune and stromal cell subpopulations of breast cancer identified in our study could be potential therapeutic targets. Taken together, Our findings lay the foundation for the precise prognostic and therapeutic stratification of breast cancer.

## Introduction

Breast cancer is a heterogeneous disease based on transcriptomic profiles and genomic aberrations 1,2. Intratumoral heterogeneity is a major contributor to disease progression, treatment resistance, and tumor recurrence. The heterogeneity of breast cancer has been witnessed in the past few decades. For example, the PAM50 intrinsic gene set classified breast tumors into five molecular subtypes with distinct biological features and clinical manifestations: luminal-A (Lum-A), luminal-B (Lum-B), HER2^+^, basal-like and normal-like 3,4. Recently, the amalgamation of precise histological examination and molecular analyses in The Cancer Genome Atlas (TCGA) breast cancer dataset unveiled 12 distinct consensus subgroups, each characterized by unique molecular signatures, particularly for less common histological types 5. Although significant progress has been made regarding the intratumoral heterogeneity of breast cancer, subtype-specific therapeutic targets still remain an unmet need to reduce disease relapse and drug resistance.

The tumorous properties specifically coordinate the traits of diverse stem or progenitor antecedents. The genetic and epigenetic programs of the cell progenitors contribute to cell-type heterogeneity by generating developmental plasticity or unique cell identities. The perturbers, including loss of tumor suppressors, oncogene activation and exogenous stimuli, may potentially reprogram the state of tumor cells throughout tumorigenesis and tumor progression. The mammary gland is a unique organ that undergoes embryonic and postnatal alterations in response to puberty and pregnancy. Recent studies have proposed that normal mammary epithelial cells mainly comprise basal/myoepithelial (BM), luminal progenitor (LP), and mature luminal (ML) subpopulations 6. Molecular profiling has laid a crucial groundwork for comprehending the etiology of breast cancer, as the intrinsic subtypes exhibit a noteworthy resemblance to normal cells within the mammary stem cell hierarchy 7. It has been robustly proved that the basal-like subtype of breast tumors carrying the *BRCA1* mutation may originate from LP but not BM cells as described by hereditary patterns 7,8. The molecular parallels imply that distinct mammary epithelial cells act as the cell-of-origin for malignant transformation across subtypes.

Single-cell RNA sequencing (scRNA-seq) analysis of breast cancer has revealed novel insights into the heterogeneity among different subtypes and the complex cellular ecosystems comprising tumor cells interspersed with immune and stromal cells 1,9. Our previous single-cell and spatially resolved analysis indicated the presence of diverse phenotypes within individual tumors by detecting the intrinsic subtyping of single malignant cells 2. Considering the hybridity of bulk sequencing, gene signatures derived from bulk RNA-seq analyses could not always accurately reflect tumor intrinsic subtype and related phenotype. Further single-cell resolved analyses on malignant cells and reference normal epithelial cells are essential for a deeper understanding of tumor cell-of-origin patterns and cellular state alterations during the specification of breast cancer subtype.

In the present study, we systematically crafted an extensive single-cell atlas of human normal and cancerous breast tissues to scrutinize tumor origins. By contrasting breast malignant cells against reference normal epithelial cells at single-cell level, we deconvoluted bulk tumor transcriptome data and uncovered intense tumor cell diversity. We agnostically profiled malignant subtypes depending on lineage specification in mammary compositions, especially a LP subtype breast cancer linked with significantly inferior prognosis but sensitivity to neoadjuvant chemotherapy (NAC), PARP inhibitors (PARPi) and immunotherapy. Performing multi-omics analyses and both *in vitro* and *in vivo* experiments, we identified PLK1 as an underlying major player in regulating chromosomal instability and a potential therapeutic target within LP subtype breast cancer. Additionally, we constructed a comprehensive single-cell atlas of breast cancer, which enabled identification of diverse novel immune and stromal cell subpopulations, including an innate lymphoid cell 3 (ILC3) subpopulation associated with favorable prognosis, CKB^+^ macrophages related to unfavorable outcomes and resistance to immune checkpoint blockades (ICBs), and a range of heterogeneous endothelial cell subsets implicated in patient prognoses and therapy responses. Collectively, this comprehensive repertoire of breast cancer provided a consummate cellular landscape and insights into patient stratification based on cancer cell-of-origin patterns.

## Materials and methods

### Tissue specimens

Paraffin-embedded tissue microarrays (tissue microarray with 219 specimens with invasive breast cancer, Hubei Cancer Hospital, China) were used for immunohistochemistry (IHC) staining. Informed consent was obtained from every participant, and the study received approval from the hospital's Ethics Committee. Slides were rigorously examined independently and blindly by two investigators. Low-quality images were dropped out for further statistical analysis.

### Cell lines and cell culture

SUM-149PT cells were cultured in Ham's F12 (Thermo Fisher, Grand Island, USA), with 10% FBS. MCF-7 cells were cultured in DMEM (Thermo Fisher, Grand Island, USA) with 10% FBS.

### Cell transfection and treatment

All transfections for plasmids and siRNAs were performed using Lipofectamine 2000 (Invitrogen, California, USA) according to the manufacturer's instructions. All chemically synthesized siRNAs were purchased from Ribobio (Guangzhou, China) and transfected at 50 nM final concentration. The siRNA target sequences for the human *PLK1* gene are 5'-CAACCAAAGTCGAATATGA-3' and 5'-GCTCTTCAATGACTCAACA-3'. The siRNA target sequences for the human *TPX2* gene are 5'-GTTTGATTCTCGAGACAAA-3' and 5'-GGAGAGAACUGGUGCAUAA-3'. The siRNA target sequences for the human *CDK1* gene are 5'-CCATGGATCTGAAGAAATA-3' and 5'-GTCAAGTGGTAGCCATGAA-3'. The siRNA target sequences for the human *AURKA* gene are 5'-GGCAACCAGTGTACCTCAT-3' and 5'-ATTCTTCCCAGCGCGTTCC-3'. PLK1 inhibitor volasertib (Cat# HY-12137) were purchased from MCE (New Jersey, USA).

### Stable knockdown of PLK1

The pSIH1-H1-puro shRNA vector was used to express PLK1 and luciferase(Luc) shRNAs. The shRNA target sequences were listed as follows: *PLK1* shRNA#1, 5'-CAACCAAAGTCGAATATGA-3'; *PLK1* shRNA#2, 5'-GCTCTTCAATGACTCAACA-3'; Luc shRNA, 5'-CUUACGCUGAGUACUUCGA-3'; SUM-149PT cells were infected with lentivirus. Stable populations were selected using 1 to 2 μg/mL puromycin. The knockdown effect was evaluated by quantitative real-time PCR (RT-qPCR).

### RT-qPCR

Total mRNA from cells was extracted by TRIzol reagent (Invitrogen, 15596026, CA, USA). A reverse transcription assay was performed by the HiScript II Q RT SuperMix for qPCR (+gDNA wiper) kit (Vazyme, R223-01, Nan Jing, China) to obtain the cDNA, and then SYBR Green Select Master Mix (Applied Biosystems, 4472908, NY, USA) was used to quantify* PLK1, TPX2, CDK1, AURKA* and 18S mRNA expression on the ABI-7900HT (Applied Biosystems). Primers were listed as follows: (Human) *PLK1*: forward 5'-GACAAGTACGGCCTTGGGTA-3', reverse 5'- TGCAGGCTGTCACCATCATT-3'; *TPX2*: forward 5'- GGCCTTTCTGGTTCTCTAGTTC-3', reverse 5'-TTGCCTTATGCACCAGTTCTC-3'; *CDK1*: forward 5'-AGCCGGGATCTACCATACCC-3', reverse 5'-CAACTCCATAGGTACCTTCTCCA-3', *AURKA*: forward 5'- TGGCGGAGCGTCAAGTC-3', reverse 5'-CAATGGAGTGAGACCCTCTAGC-3', 18S: forward 5'-CTCAACACGGGAAACCTCAC-3', reverse 5'- CGCTCCACCAACTAAGAACG-3'.

### Sulforhodamine B assays

Cells were spread in 96-well plates, treated with drugs for a certain period of time, and then fixed with 10% trichloroacetic acid at 4 °C overnight. Afterward, the plates were gently washed and allowed to dry, followed by staining with 100 μL SRB stain (0.4% in 1% acetic acid) for 30 minutes, and the unbound dye was washed with 1% acetic acid. The plates were allowed to dry, and the stains were dissolved by adding 100 μL Tris buffer (10 mM, pH 10.5). Absorbance was measured at a wavelength of 530 nm using a microplate reader (Infinite M200Pro, Tecan).

### Apoptosis analysis

An Annexin Detection Kit (1133534, BD Pharmingen) was used to measure apoptosis. After the cells were treated with a D43 concentration gradient, floating and adherent cells were collected together and centrifuged at 500 × g for 5 minutes. The cells were washed with PBS and then stained with FITC/Annexin V and propidium iodide following the manufacturer's instructions. The proportion of apoptotic cells was determined by flow cytometry. The assays were repeated at least three times.

### Genomic instability analysis and cytogenetics

Exponentially growing cultures were treated with colcemid (0.04 μg/ml) for 2-3 h. Cells were harvested, incubated in hypotonic solution (0.075 M KCl) and fixed in methanol-acetic acid (3:1 vol/vol), and stained with 4% Giemsa solution or DAPI 0.5 mg/ml (1:250). The slides were analyzed for chromosomal aberrations. Images were captured and analyzed using a Nikon 80i Microscope and karyotyping software (Applied Spectral Imaging, Inc.).

### Animal experimentation

We purchased 5- to 6-week-old female BALB/c nude mice from SLACCAS (Changsha, China). The animal protocol was approved by the animal ethics committee of Kunming Institute of Zoology, CAS. Nude mice were randomly distributed into three groups (shLuc, shPLK1 1#, shPLK1 2#). Three million SUM-149PT cells resuspended in Matrigel (BD Biosciences; 1:1 diluted with 2% FBS in PBS) were injected into the third pair of mammary gland fad pads. For the PLK1 inhibitor volasertib experiment, two million wild-type SUM-149PT cells were implanted into the mammary fat pads of the mice. when the tumor volume reached approximately 50 mm^3^, the nude mice were randomly assigned to the control and treatment groups. The control group was given vehicle alone, and the treatment group received PLK1 inhibitor volasertib (12.5 or 25 mg/kg) alone via gavage every four days for 20 days. The tumor volume was calculated as follows: tumor volume was calculated by the formula: (π×length×width^2^)/6.

### Immunohistochemistry

Six series of tissue microarray were used to detect PSAT1 (Proteintech, 10501-1-AP, 1:200), ER (Proteintech, 21244-1-AP, 1:200), CK14 (Servicebio, GB11803, 1:200), CKB (Proteintech, 66714-1-IG, 1:500) and CA4 (Proteintech, 13931-1-AP, 1:200). Immunohistochemical staining was performed as follows: deparaffinization, antigen retrieval, blocking endogenous peroxidase (3% hydrogen peroxide solution, room temperature, out of light for 25 minutes), serum block (3% bovine serum albumin, room temperature, 30 minutes), primary antibodies were incubated overnight at 4 °C, and horseradish peroxidase (HRP)-conjugated for 50 minutes at room temperature. Staining was visualized with DAB and time controlled under a microscope. Finally, nuclear counterstaining was performed using hematoxylin for approximately 3 minutes. The staining results were scored by two independent pathologists as follows: the protein expression levels of CKB and CA4 was described by the average number of positive macrophages and endothelial cells (ECs) respectively, from five fields at a magnification of ×400 using Image-Pro Plus, while the protein expression levels of PAST1, ER and CK14 were described by the percentage of high positive cells calculated by ImageJ software. In addition, surv_cutpoint function was used to determine the best cut-off values of all protein expression levels with respect to the survival rate.

### Single-cell RNA-Seq datasets collected in this study

**Figure 1:**
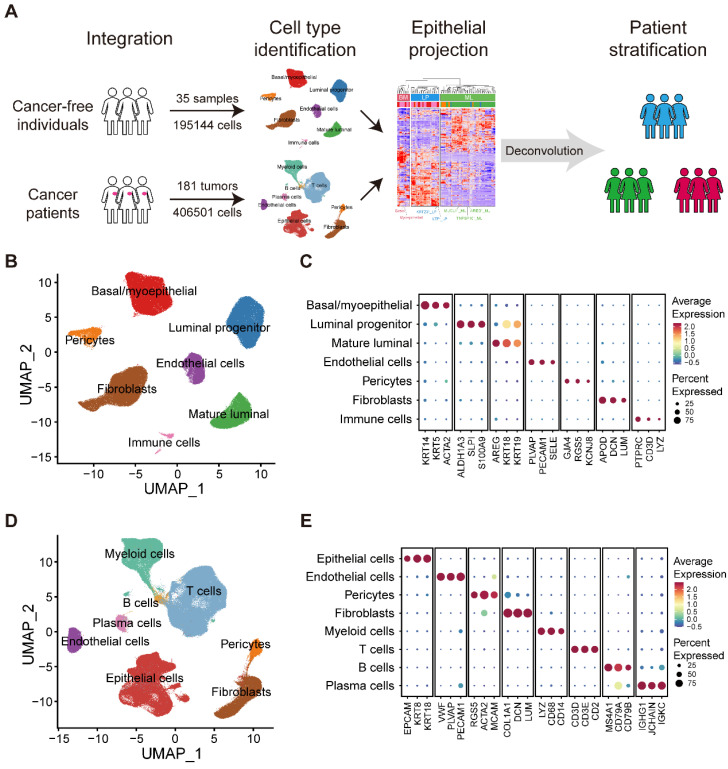
** Large-scale integrated cellular landscape of human normal and cancerous breasts.** (A) Schematic diagram of the study design and analysis. (B) Integrated analysis of 195,144 cells from 35 normal breast tissues. (C) Bubble heatmap showing expression levels of selected signature genes in normal breast tissues. Dot size indicates fraction of expressing cells, colored based on average normalized expression levels. (D) Integrated analysis of 406,501 cells from 181 primary breast tumors. (E) Bubble heatmap showing expression levels of selected signature genes in breast tumors. Dot size indicates fraction of expressing cells, colored based on average normalized expression levels.

We obtained scRNA-seq data on normal breasts in 35 samples from 35 individuals and breast tumors in 181 samples from 134 patients diagnosed with breast cancer (**Figure 1A**; **Table S1**). Data from normal breasts including total cells or only epithelial cells were obtained from published datasets. To supplement the publicly available data studying tumor-infiltrating immune cells, we collected several additional datasets on purified immune cells.

### Analysis of scRNA-seq data

We applied each sample separately to perform unsupervised clustering of the single cells using the read count matrix as input by Seurat package (v4.1.1) in R (v4.1.3). The quality control applied to cells was mainly based on the number of detected genes and proportion of mitochondrial gene count per cell. Firstly, cells with fewer than 200 detected genes and cells with over than 15% mitochondrial gene count were filtered. To avoid unexpected noise, genes detected in less than 3 cells were excluded from the downstream analysis. The potential contamination or doublet cells were removed by dropping the clusters or by filtering out cells with high average expression of the signature genes of the contaminating cell types. To reduce the dropout impact on downstream analysis, we filtered out cells harboring obviously low UMI count. Finally, 195,144 cells from normal breasts and 406,501 cells from breast cancer samples remained and were enrolled in downstream analysis. To correct the batch effects, data integration was performed by mutual nearest neighbor (MNN) method via Seurat-Wrappers package (v0.3.0). We next performed dimension reduction clustering and differential expression analysis following the Seurat-guided tutorial. The principal component analysis (PCA) and uniform manifold approximation and projection (UMAP) dimension reduction were conducted with the top 15 principal components. The FindAllMarker function was used to identify preferentially expressed genes between diverse clusters. The S phase score and G2M phase score were calculated through function CellCycleScoring, and the naïve, cytotoxic, exhausted and Treg signature scores of T cells were calculated with function AddModuleScore.

### Analysis of bulk RNA-seq data

Normalized METABRIC and TCGA expression matrices and clinical information were obtained from cBioPortal website (http://www.cbioportal.org/). Transcriptome data and clinicopathological information of the NAC dataset (GSE163882) and I-SPY2 dataset (GSE194040) were obtained from Gene Expression Omnibus (GEO, https://www.ncbi.nlm.nih.gov/geo/).

### Mapping of tumor cells in three normal epithelial subsets

We firstly transformed the normal epithelial cell expression matrix into seven pseudobulk profiles according to the aforementioned minor subsets. The top 100 positively expressed markers of each normal epithelial minor subset with redundancy elimination were regarded as gene sets accounting for the heterogeneity of normal epithelial cells (**Table S2**). Subsequently, we constructed 115 pseudobulk profiles after re-clustering 113,254 epithelial cell from primary breast tumors (**Figure S2B**). The top 1% variable genes based on variance analysis of these 115 pseudobulk profiles were considered as gene sets deciphering the heterogeneity among tumor cells (**Table S3**). We noted a high overlap between these two gene sets and extracted their intersection which included 182 genes for subsequent tumor-normal projection (**Table S4**). Then we combined these seven normal epithelial pseudobulk profiles and 115 tumor pseudobulk profiles, and corrected the batch effects in the 182 meta-genes space. Through unsupervised clustering of these 122 pseudobulk profiles, we revealed three major groups represented by seven subsets of normal epithelial cells, potentially indicating different cell-of-origin patterns.

### Deconvolution of bulk transcriptome data

On the basis of our integrated scRNA-seq data and 115 epithelial pseudobulk profiles, we initially devoted to selecting the marker genes to represent three breast epithelial subtypes based on the Spearman correlation coefficient shown on **Figure S3A**. Specifically, the top 150 genes correlated with the composition of ML subtype were defined as the marker genes of ML subtype. However, we observed it is ambiguous to identified the marker genes of LP and BM subtypes based on the correlation coefficient solely. Therefore, we calculated the distance between each point and some certain flag in the coordinate axis of **Figure S3A**. The D1 distance was calculated as: D1 = x^2^+(y-1)^2^. The D2 distance was calculated as: D2 = (x-1)^2^+(y-1)^2^. The D3 distance was calculated as: D3 = (x-1)^2^+y^2^. Finally, the marker genes of LP subtype were defined as the union of top 150 genes of D1 and D2 distance. The marker genes of BM subtype were defined as the union of top 150 genes of D2 and D3 distance. Next, DWLS 10 was performed to deconvolute predicted cell fractions from the METABRIC dataset based on the above identified lineage markers. The deconvolution analysis generated scores of three breast epithelial subtype in each tumor sample (**Figure 3A**). This score of three breast epithelial subtype could be interpreted as the proportion of the corresponding cancer-cell-of-origin patterns in a tumor sample.

### CNV estimation

Initial CNVs for each region of individual cells were estimated via infercnv package (v1.10.1) 11. The CNV score of each cell was calculated as the variance of CNV_region_.

### Functional annotation of genes

Gene Set variation Analysis (GSVA) were performed to investigate the pathway activities by GSVA package from Bioconductor 12. The hallmark gene sets we investigated were downloaded from MSigDB. The pathways with high difference in activity scores were selected by LIMMA package. Gene Ontology (GO) analysis was performed to annotate given gene lists of interest using the R package clusterProfiler (v4.0.5).

### Calculation of LP signature score in TCGA cohort

The correlation between genes and LP proportion in METABRIC cohort was analyzed via Spearman correlation analysis. The top and bottom 100 genes were defined as the LP-positive and LP-negative associated markers, respectively. To obtain the LP signature score, we calculated the positive and negative signature score by GSVA based on the LP-positive and LP-negative associated markers in TCGA dataset. Then, the final LP signature score (denoted as the LP score) was defined as the difference value of LP positive and negative signature score. In TCGA cohort, samples with the LP score higher than the median were referred to as LP-high tumors.

### Identification of potential key factors involved in chromosomal instability within LP subtype breast cancer

For scRNA-seq data, the CNV scores of LP group malignant epithelial cells were calculated by inferCNV algorithm as mentioned above. Genes that were significantly differentially expressed in the top 25% of the highest scoring LP group cancer cells compared to the bottom 25% of the lowest scoring cells were considered as candidate genes. For bulk RNA-seq data, genes with copy number alterations (CNA) significantly correlated with the LP signature score in TCGA breast cancer cohort were considered as target genes. For Perturb-seq data developed and analyzed previously 13, CRISPR-based screens with scRNA-seq readouts identified genes inducing remarkable chromosomal instability as the target genes. The rank score was defined as the summation of the rankings of the 260 genes that intersected across the three omics data rankings. In other words, a higher ranking corresponds to a higher rank score.

### Definition of cancer cell states, and mutation and immune signatures

A catalog log of gene modules defining 16 recurrent cancer cell states was proposed by Barkley et al. 14, and provided in **Table S5**. The mutation signatures of TCGA samples were referenced by previous studies 15,16. The published signature gene lists for naïve, cytotoxic, exhausted T cell and Treg, as well as DC cell activation, migration and tolerogenic had been previously described 17 and showed in **Table S6** and **S7**. Besides, the M1-like and M2-like tumor-associated macrophage (TAM) signatures were previously described 18 and listed in **Table S8**.

### SCENIC analysis

The SCENIC analysis was performed by using the pyscenic (v0.11.2) 19 and hg19-tss-centered-10kb-10species databases for RcisTarget, GRNboost, and AUCell. The input matrix was the normalized expression matrix that was from Seurat.

### Survival analysis

To model the effect of LP-derived cancer cell proportion on breast cancer patient survival, we performed multivariable Cox regression on METABRIC datasat. Signature scores of diverse identified cell subpopulations were calculated as the GSVA scores of the top 50 significantly expressed genes. Kaplan-Meier curves were plotted to show the survival difference using R package survival and survminer. We performed the survival analysis by focusing the ten-year survival after disease diagnosis. Patient stratification was based on the best cut-off calculated by surv_cutpoint function. Log-rank test statistics was conducted to assess the significance between two groups.

### Assessing the heterogeneity of single cell populations

To compare the heterogeneity of cell subpopulations in our study, we used ROGUE 20, which was an entropy-based universal metric for assessing the purity of single cell population. We scaled the ROGUE index to between zero and one. One represented a completely pure subtype with no significant genes and zero represented the most heterogeneous state of a population.

### Statistical analyses

All statistical analyses and graphs were done in R (v4.1.3). Wilcoxon rank sum test was performed to assess significance in signature score analysis and cell proportion differences analysis. Kaplan-Meier survival data were analyzed via two-sided log-rank test. Adjusted p-values above 0.05 were regarded as not significant. Benjamini-Hochberg false discovery rate (FDR) correction was performed at a p-value of 0.05 for multiple comparison correction (proportions for subtypes of scRNA data). The p-values in the figures were reported using the following symbols: *P < 0.05, **P < 0.01, ***P < 0.001, ****P < 0.0001.

## Results

### Large-scale integrated cellular landscape of human normal and cancerous breasts

To generate a comprehensive and single-cell resolved transcriptional atlas of normal and cancerous breast tissues, we collected and analyzed scRNA-seq data of 35 normal breast tissues and 181 primary breast cancer samples from public datasets (**Figure 1A and Table S1**) 1,21-29. After quality control and data preprocessing, a total of 195,144 high-quality cells from normal breast tissues and 406,501 high-quality cells from primary breast cancer samples were included in our analysis (**Figure 1A**). To characterize the cellular components of normal breast tissues, we performed unsupervised graph-based clustering on all cells after correcting the batch effects across different datasets (**Figure 1B**). All major cell types, including three epithelial subsets (BM, LP and ML), immune cells, endothelial cells, fibroblasts and pericytes, were annotated based on canonical cell markers and visualized by UMAP (**Figure 1B-C and Figure S1A**). For the tumor compartment, apart from epithelial cells, endothelial cells, fibroblasts and pericytes, we identified abundant immune subsets, including T cells, B cells, plasma cells and myeloid cells, with unique cellular identities (**Figure 1D-E and Figure S1B**).

### Alignment of breast cancer cells to normal breast epithelial cell subtypes

**Figure 2:**
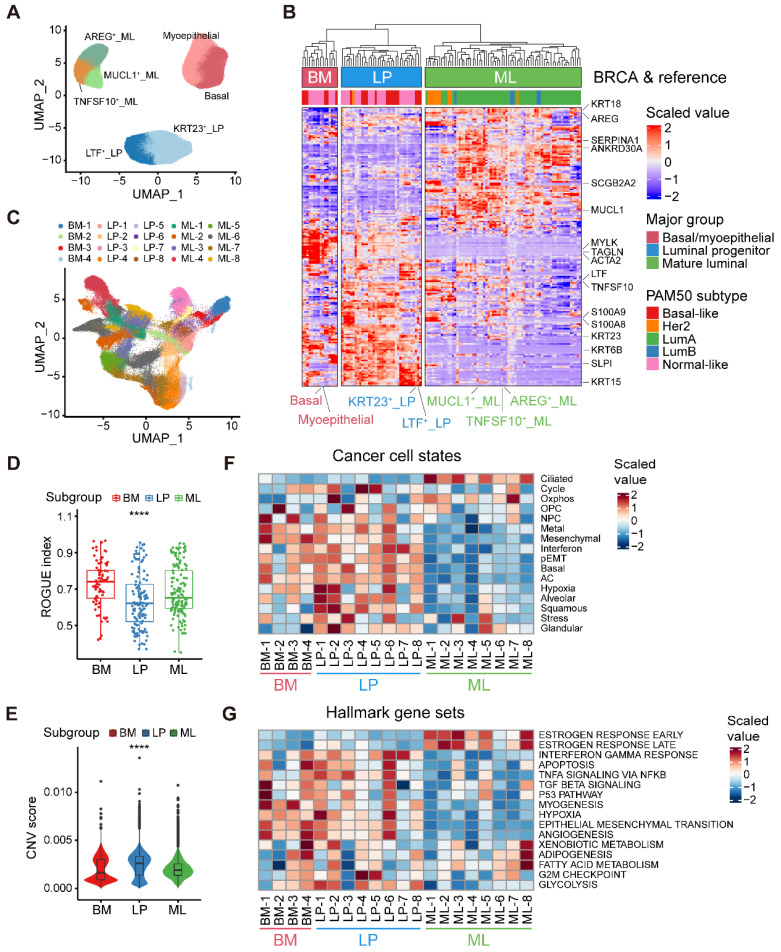
** Alignment of breast cancer cells to normal breast epithelial cell subtypes.** (A) UMAP plot showing the major lineages of epithelial cells in normal breast tissues. (B) Clustering of 115 breast tumor pseudobulk profiles combined with seven reference normal mammary epithelial pseudobulk profiles showing three major lineages based on the expression of common signature genes. (C) UMAP plot showing 20 breast tumor subpopulations within three major lineages of breast cancer cells. (D) Boxplot showing cell purity for breast cancer cells within three major lineages. Kruskal-Wallis test. (E) Violin plot showing distributions of CNV scores among breast cancer cells from three major lineages. Kruskal-Wallis test. (F) Heatmap showing different expression patterns of 16 recurrent cancer cell gene modules among 20 breast tumor subpopulations. (G) Heatmap showing different expression patterns of hallmark gene sets among 20 breast tumor subpopulations.

Our aforementioned analyses showed three epithelial major clusters in normal breasts, namely the BM, LP, and ML subpopulations. To further decipher the compositions of normal breast epithelial cells, we reclustered 117,729 high-quality normal epithelial cells and identified seven minor subpopulations based on the expression of diverse gene signatures (**Figure 2A and Figure S2A**). Within the BM cells, we revealed two distinct cell subsets, one exhibiting remarkable expression of specific epithelial keratin and integrin members (*KRT5* and *ITGB1*; basal) and the other with featured markers associated with myoepithelial cell differentiation (*ACTA2*, *TAGLN* and *MYLK*; myoepithelial). The LP cells covered a subset expressing markers related to lactotransferrin and certain members of the S100 family (*LTF*, *S100A8* and *S100A9*; LTF^+^ LP), while the other showed specific expression of markers linked to stemness and certain specific epithelial keratins (*ALDH1A3*, *KRT15* and *KRT23*; KRT23^+^ LP) 30. Luminal-specific marker genes such as *KRT8* and *KRT18*, were both expressed in LP and ML cells (**Figure S2A**). Within the ML cells, we identified three distinct cell clusters, featured by the expression of the epidermal growth factor (EGF)-like molecule amphiregulin (*AREG*; AREG^+^ ML), member of the tumor necrosis factor (TNF) ligand family (*TNFSF10*; TNFSF10^+^ ML), and mucin proteins (*MUCL1*; MUCL1^+^ ML).

Next, to explore the correlation between epithelial cell clusters from normal and cancerous breasts, we contrasted malignant cells against reference normal mammary epithelial cells by evaluating the homology of signature gene expression patterns. To be specific, we initially calculated a total of 115 pseudobulk profiles after reclustering 113,254 epithelial cell from primary breast tumors, as well as seven reference pseudobulk profiles from normal breast tissues (**Figure 2A and Figure S2B**). Unsupervised clustering uncovered that neoplastic cells could be divided into three major groups (BM group, LP group and ML group), potentially indicating distinct cancer cell-of-origin patterns (**Figure 2B**). To further validate the accuracy of the cancer cell-of-origin patterns based on unsupervised clustering, we performed PCA analysis and found concordant grouping with the above clustering (**Figure S2C**). These three major groups harbored diverse canonical epithelial markers, further supporting the clustering on account of cancer cell-of-origin patterns. For example, special markers linked with myoepithelial cell function (*ACTA2*, *TAGLN*, and *MYLK*) were significantly expressed in the BM group from both normal and malignant epithelial cells (**Figure 2B**). Furthermore, we investigated the association between PAM50 which was a 50-gene signature developed to define breast cancer molecular subtypes, and the clustering based on our proposed cancer cell-of-origin patterns (**Figure 2B**). Basal-like and normal-like subtype tumors were exclusively found in the BM and LP groups, potentially as the origins of hormone-negative tumors (**Figure 2B**). In addition, all Lum-A and Lum-B tumors belonged to the ML group, indicating that ML cells were the origins of hormone-positive tumors (**Figure 2B**). Although HER2^+^ tumors were predominantly distributed in the ML group, we found that a small subset of HER2^+^ tumors was derived from the LP group (**Figure 2B**). Additionally, we could classify these single-cell breast cancer samples into diverse molecular subtypes according to the proportion of tumor cells with diverse origins (**Figure S2D**).

Furthermore, we examined the internal heterogeneity within each group. In the BM, LP, and ML groups, consensus clustering revealed four, eight, and eight tumor cell clusters with similar gene expression, respectively (**Figure S2E-G**). Based on cancer cell-of-origin patterns as well as internal heterogeneity, the entirety was divided into 20 subpopulations and visualized via UMAP (**Figure 2C**). Using ROGUE analysis, a general metric for evaluating the purity of a single cell population based on entropy, we observed that cancer cells in LP group exhibited the highest heterogeneity among three major subgroups (**Figure 2D**). To understand the genomic alteration in these neoplastic clusters, we estimated single-cell copy number variants (CNVs) by the inferCNV algorithm, and the results showed that malignant cells in LP group exhibited remarkably high CNV levels (**Figure 2E**). Moreover, in order to determine the biological properties of these subpopulations, we then sought to determine the recurrent states of cancer cells as previously described (**Figure 2F and Table S5**) 14. The mesenchymal, basal, and partial epithelial-mesenchymal transition (pEMT) modules were highly scored in the BM and LP groups but negatively expressed in the ML group (**Figure 2F**). The LP-original cancer cells had higher scores of the interferon, squamous, and glandular modules (**Figure 2F**). In addition, elevated levels of oxidative phosphorylation and ciliated modules were exhibited in ML group cancer cells (**Figure 2F**). Regarding the internal heterogeneity within each group, the LP-1, LP-2, and LP-6 subgroups showed elevated hypoxia scores, while LP-2, LP-4 and LP-5 had higher cell cycle scores than the other LP tumor groups (**Figure 2F**). Furthermore, we investigated the functional heterogeneity in these subpopulations using the hallmark gene sets. As expected, expression of estrogen response-related genes were enriched in the ML subtypes (**Figure 2G**). In addition, the interferon gamma response and EMT were enriched in most of the BM and LP subclusters (**Figure 2G**). Apparently, the BM-1 subset showed a significant enrichment of the TGF-β signaling, p53 pathway and myogenesis. Upregulation of adipogenesis and fatty acid metabolism was found in the ML-8 subpopulation (**Figure 2G**). In summary, we identified three molecular subtypes of breast cancer cells based on cancer cell-of-origin patterns and meticulously characterized the cancer cell state and functional heterogeneity within each molecular subtype.

### The molecular and clinical characteristics of LP subtype breast cancer

**Figure 3:**
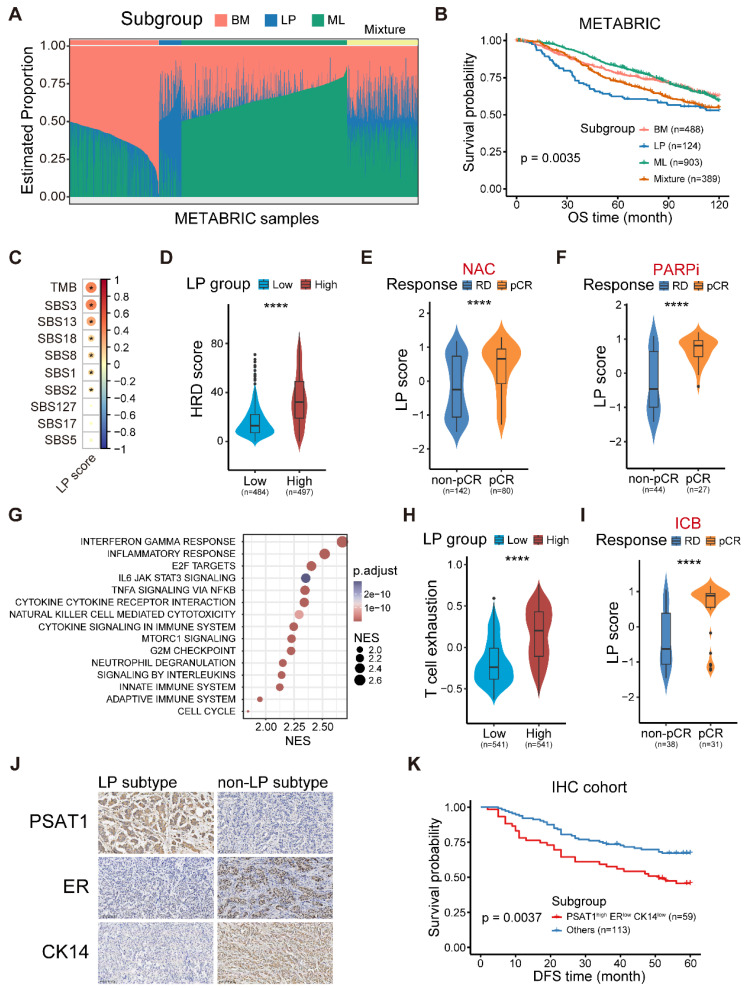
** The molecular and clinical characteristics of LP subtype breast cancer.** (A) Stacked bar plot showing the deconvolution result of breast tumors from the METABRIC cohort. Colors of the bars denote three cell lineages as shown in the legend. The y axis stands for the proportion of each cell lineage in a given bulk tumor sample. Within the x axis, each column represents one tumor case. The annotation bar above denotes the molecular subtypes of bulk tumors that are defined by the dominant cell lineage within each tumor, where yellow represents the mixture of multiple cell lineages. (B) Kaplan-Meier plot showing worse clinical outcome in LP subtype patients within the METABRIC cohort. *P* value is calculated using the log-rank test. (C) Spearman correlation between LP score and diverse mutational signatures. Correlations with *P* < 0.05 are marked with an asterisk. (D) Violin plot comparing the HRD score between LP-low and LP-high breast tumors in TCGA. Unpaired two-sided Wilcoxon test. (E) Violin plot comparing the expression level of LP score of breast tumors with different responses to NAC treatment. Unpaired two-sided Wilcoxon test. (F) Violin plot comparing the expression level of LP score of breast tumors with different responses to PARP inhibitor treatment in the I-SPY2 cohort. Unpaired two-sided Wilcoxon test. (G) Bubble heatmap showing up-regulated pathways enriched in breast tumors with high LP proportion via GSEA. Dot size indicates the normalized enrichment score (NES), colored based on the adjusted *P* value. (H) Violin plot comparing the T cell exhausted signature score between LP-low and LP-high breast tumors in TCGA. Unpaired two-sided Wilcoxon test. (I) Violin plot comparing the expression level of LP score of breast cancer patients with different responses to anti-PD1 treatment. Unpaired two-sided Wilcoxon test. (J) Immunohistochemistry of breast tissue microarray for PSAT1, ER and CK14. (K) LP subtype of breast cancer patients with PSAT1^high^/ER^low^/CK14^low^ were associated with poor DFS. *P* value is calculated using the log-rank test.

To determine the clinical relevance of the established cancer cell-of-origin patterns, we first performed deconvolution analysis in the METABRIC cohort to compute the composition of three subtypes within each tumor (**Figure S3A**). The deconvoluted samples presented remarkable intratumoral heterogeneity according to the inferred proportion of tumor subtypes (**Figure 3A**) 10. The LP subtype was significantly associated with an unfavorable outcome, and this association was independent of age, tumor grade, tumor size, tumor stage, tumor mutation burden (TMB) and cell cycle score (**Figure 3B and Figure S3B**). Regarding clinical features, we found the proportion of the LP cells was higher in young patients, and the cases with LP-dominant subtype were positively associated with higher tumor grade and lymphoid node metastasis (LNM) (**Figure S3C-E**). Moreover, the tumors in LP subtype were characterized as an elevated cell cycle score, suggesting that the LP-predominated tumors might exhibit rapid proliferation (**Figure S3F**).

Furthermore, we generated a robust meta-gene expression signature of the LP subtype as a surrogate for their cellular abundance, denoted thereafter as the LP score (**Table S2-S4**; **Materials and methods**). The patients with a higher level of LP score showed poor clinical outcomes in the TCGA cohort, coherent with the observation in the METABRIC cohort (**Figure S4A**). Given the high CNV burden in LP-derivating cancer cells, we further explored the relationship between the LP-dominated malignancies and genomic alteration profiles in TCGA dataset.

Consistent with the raised CNV burden inferred in single-cell analysis, tumors in LP subtype demonstrated higher TMB level, particularly those of COSMIC mutational signature SBS3 (associated with homologous recombination defect, HRD) and SBS13, but not SBS2 (both associated with APOBEC-mediated deamination) (**Figure 3C**) 15. HRD-related complex genomic alteration events (HRD score) were also significantly elevated in breast tumors with higher LP scores (LP-high) (**Figure 3D**), as well as HRD-related copy number signature (CN17) and chromosomal instability signatures (CX2, 3, and 5) (**Figure S4B-C**) 31,32. UNG is specifically involved in the formation of SBS13-type mutations. An elevated level of *UNG* expression as well as SBS13 events (kataegis and omikli) was found in LP-high tumors (**Figure S4D-E**). Moreover, LP-high tumors had a higher incidence of whole genome duplication (WGD), and presented an increase of aneuploidy and genomic abnormity (**Figure S4F-H**). These findings echoed the entropy-based single-cell diversity observation by the ROGUE analysis (**Figure 2D**). Given the genomic instability of LP-high tumors, we further explored their therapeutic significance, especially regarding the clinical relevance in NAC and synthetic lethality like PARPi. As expected, increased expression levels of the p53 deficient-related and PARPi response-related meta-gene signatures were found in LP-high tumors (**Figure S4I**). Notably, LP-high patients were sensitive to NAC and PARPi treatment (**Figure 3E-F and Figure S4J**) 33. Taken together, these findings suggested the LP subtype breast cancer exhibited higher TMB and genomic instability, but sensitivity to NAC and PARPi treatment.

We next explored the functional and immune characteristics of LP subtype breast cancer in clinical cohorts. Gene set enrichment analysis (GSEA) showed that the transcriptional characteristics of the LP subtype were enriched in the interferon gamma response, inflammatory response, cytokine-cytokine receptor interaction, and innate immune system pathways (**Figure 3G**). In addition, richness of T-cell receptor (TCR) and B-cell receptor (BCR), as well as single nucleotide variant (SNV) and insertion-deletion (indel) neoantigens, was prominent in LP-high tumors (**Figure S5A**). Next, the profiles of the immune microenvironment in LP-high tumors were investigated in both the METABRIC and TCGA datasets (**Figure S5B**). The LP-high patients showed an increased abundance of CD8^+^ T cells, follicular helper T cells, and M1 macrophages (**Figure S5B**). However, LP-high tumors exhibited an elevated expression level of T cell exhaustion signature score and various immunosuppressive checkpoints (**Figure 3H and Figure S5C**). Moreover, we demonstrated that the LP subtype patients were more likely to achieve pCR after ICB combined with NAC in the I-SPY2 cohort (**Figure 3I and Figure S5D**). Overall, the LP subtype was associated with dysfunctional immune characteristics and sensitivity to immunotherapy.

Finally, we sought to explore candidate markers of the LP subtype of breast cancer for clinical diagnosis and therapeutic decisions. Spearman correlation analysis at both the transcriptional and protein levels in TCGA dataset revealed that phosphoserine aminotransferase 1 (PAST1) was the optimal marker for the LP subtype (**Figure S5E-F and Table S9**). To determine whether a relationship exists between the PSAT1 status and clinical progression, IHC staining was applied to our breast cancer tissue microarrays. The results showed that PSAT1 staining was predominantly cytoplasmic (**Figure 3J**), and PSAT1 overexpression indicated a poor disease-free survival (DFS) (**Figure S5G**). Similarly, we selected ER to represent the ML subtype and CK14 to represent the BM subtype based on the subtype-specific expression profiles across diverse subtypes (**Figure S5F**, **H-I**). Using these biomarkers, the LP molecular subtype was defined as PSAT1^high^/ER^low^/CK14^low^ in our IHC cohort (**Figure 3J**), and the LP group revealed inferior prognosis (**Figure 3K**). In summary, we identified the LP subtype breast cancer with a significant inferior prognosis and special molecular and clinical characteristics, and further screened and validated candidate markers for clinical practice.

### Identification of potential therapeutic targets in the LP subtype breast cancer

**Figure 4:**
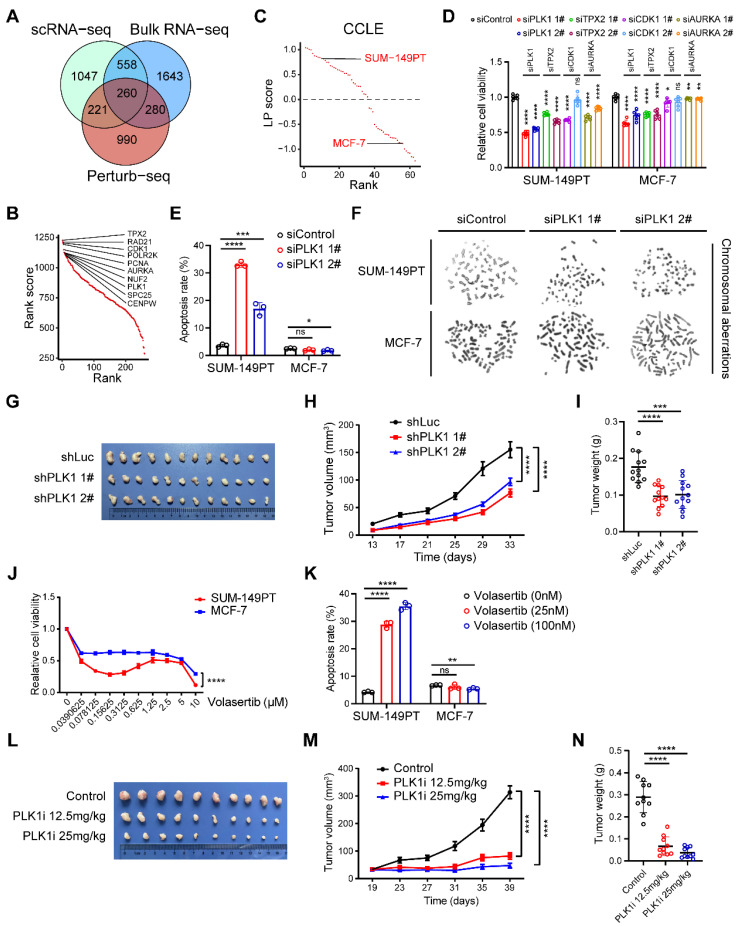
** Identification of potential therapeutic targets in the LP subtype breast cancer.** (A) Venn diagram illustrating unique and shared genes driving chromosomal instability within LP subtype breast cancer identified by scRNA-seq, bulk RNA-seq and Perturb-seq data. (B) Scatter plot showing the rank scores of 260 common genes identified by scRNA-seq, bulk RNA-seq and Perturb-seq data. The top 10 genes are highlighted. (C) Scatter plot showing the LP scores of diverse breast cancer cell lines in CCLE. The SUM-149PT and MCF-7 cell lines are highlighted. (D) Levels of relative cell viability of selected candidate genes (*PLK1*, *TPX2*, *CDK1* and *AURKA*) knockdown in the SUM-149PT and MCF-7 cells. (E) Levels of apoptosis rate of SUM-149PT and MCF-7 cells with *PLK1* knockdown. (F) Representative images of Giemsa staining of *PLK1* knockdown and control vector in the SUM-149PT and MCF-7 cells. (G) The picture of SUM-149PT tumors with *PLK1* knockdown and control vector. (H) Tumor growth curves of mice that were injected with SUM-149PT cells with *PLK1* knockdown and control vector. (I) Weight distributions of SUM-149PT tumors with *PLK1* knockdown and control vector are shown. (J) The curve showing the relative cell viability of SUM-149PT and MCF-7 cells following treatment with volasertib at various doses. (K) Levels of apoptosis rate of SUM-149PT and MCF-7 cells following treatment with volasertib at high and low doses. (L) The picture of SUM-149PT tumors following treatment with volasertib at high and low doses and control vector. (M) Tumor growth curves of mice that were injected with SUM-149PT tumors following treatment with volasertib at high and low doses and control vector. (N) Weight distributions of SUM-149PT tumors following treatment with volasertib at high and low doses and control vector.

Considering chromosomal aberration stood as a conspicuous hallmark characterizing the LP subtype of breast carcinoma, we next devoted to exploring the major factors involved in chromosomal instability as potential therapeutic targets within the LP subtype, spanning a panorama of diverse omics realms. Combining putative players derived from the integration of single-cell and bulk RNA-seq analyses within this study, alongside the genes discerned through a recent genome-scale Perturb-seq investigation (**Materials and methods**) 13, we successfully elucidated a cohort of 260 candidate genes, including *PLK1*, *TPX2*, *CDK1* and *AURKA* (**Figure 4A-B**). Moreover, we computed the LP signature score for a range of mammary carcinoma cell lines using the GSVA technique. We meticulously identified SUM-149PT as the quintessential LP-subtype mammary cancer cell line, while designating MCF-7 as the paradigmatic control non-LP mammary cancer cell line (**Figure 4C**). To screen out major factors involved in chromosomal instability within LP subtype breast cancer, we initially found that the inhibition of *PLK1*, *TPX2*, *CDK1* and *AURKA* exerted diverse impacts on the proliferation of tumor cells, as elucidated by the gradations observed (**Figure 4D and Figure S6A-D**).

To expound further, the attenuation of PLK1 emerged as the most efficacious intervention in suppressing the viability of SUM-149PT cells (**Figure 4D**). Henceforth, we elected PLK1 as the pivotal factor for elucidating the intricacies of the LP subtype. We observed that the inhibition of PLK1 substantially enhanced the apoptotic process in SUM-149PT cells, without exerting a discernible impact on MCF-7 cell apoptosis (**Figure 4E**). Cytogenetic inquiry revealed that SUM-149PT cells, upon undergoing PLK1 depletion, manifested an augmented frequency of chromosomal aberrations, encompassing phenomena such as chromosomal truncation and rupture (**Figure 4F**). This phenomenon was not discerned within MCF-7 cells subjected to analogous PLK1 suppression (**Figure 4F**). Furthermore, we proceeded to evaluate the impact of PLK1 in murine models to investigating its impact on tumor growth. Remarkably, we discerned a deceleration in tumor expansion within the PLK1-inhibited SUM-149PT cells, juxtaposed with the control vector cells (**Figure 4G-I and Figure S6E**). In addition, we determined to investigate the potential impact of the PLK1 inhibitor, volasertib, on the proliferation dynamics of both LP and non-LP subtypes within the realm of breast cancer cells. Notably, under volasertib treatment, SUM-149PT cells but not MCF-7 cells demonstrated a pronounced suppression of cellular proliferation alongside a noteworthy augmentation of apoptotic induction (**Figure 4J-K**). In the context of the SUM-149PT mice model, we observed a comparable attenuation in tumor growth subsequent to the administration of volasertib (**Figure 4L-N**). Taken together, these results propose that PLK1 potentially functions as a fundamental factor involved in chromosomal instability within the LP subtype breast cancer, thus emerging as a plausible therapeutic target warranting exploration within the domain of LP subtype breast malignancies.

### Integrated analyses of lymphocytes, natural killer (NK) cells and innate lymphoid cells (ILCs)

**Figure 5:**
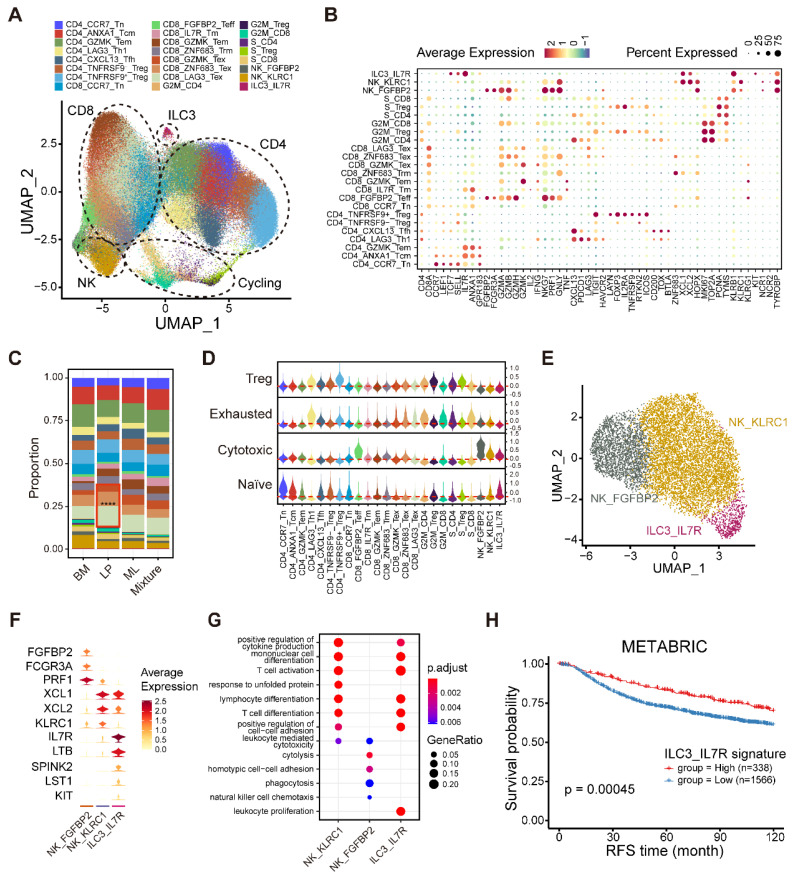
** Integrated analyses of lymphocytes, NK cells and ILCs.** (A) UMAP plot showing diverse subsets of T cells and ILCs. (B) Bubble heatmap showing expression levels of selected signature genes in T cells and ILCs. Dot size indicates fraction of expressing cells, colored based on average normalized expression levels. (C) Bar chart showing the relative proportion of major T/ILC cell types in different molecular subtypes. (D) Violin plot showing representative naïve, cytotoxic, exhausted and Treg signatures in diverse T/ILC subsets. Dashed red line denotes the median module score. (E) UMAP plot showing three main subsets of NK/ILC cells. (F) Violin plot showing expression levels of selected signature genes in NK/ILC cells. (G) GO enrichment analysis using the top 50 significantly expressed genes of each NK/ILC subset. (H) Kaplan-Meier plot showing better clinical outcome in breast cancer patients with higher composition of ILC3_IL7R subset within the METABRIC cohort. *P* value is calculated using the log-rank test.

Given the distinct immune microenvironment in different molecular subtypes of breast cancer, we next aimed to build a high-resolution immune cell landscape of breast cancer by integrating scRNA-seq technology and bioinformatics approaches. Firstly, we integrated 156,289 T cells and NK/ILCs and identified 24 clusters containing seven CD4^+^ T clusters, eight CD8^+^ T clusters, six cycling T clusters and three NK/ILCs clusters (**Figure 5A, Figure S7A-B**). We then annotated these clusters according to the expression of marker genes and functional signatures (**Figure 5B**). All major cell types were represented and manifested varying proportions in different molecular subtypes (**Figure 5C**). To explore the biological features of these clusters, we preliminarily scored the naïve, cytotoxic, exhausted and regulatory T cell signatures among diverse subpopulations (**Figure 5D and Table S6**). For example, the FGFBP2^+^ effector T (Teff) cluster and FGFBP2^+^ NK cluster revealed markedly higher cytotoxic scores than the other clusters (**Figure 5D**). In the CD4^+^ T cell compartment, we found several classical subclusters, including typical CCR7^+^ naïve T (Tn), GZMK^+^ effector memory T (Tem), ANXA1^+^ central memory T (Tcm) and FOXP3^+^TNFRSF9^+^ regulatory T (Treg) cells. We also identified a LAG3^+^ T helper 1 (Th1) cell cluster, a CXCL13^+^ T follicular helper (Tfh) cell cluster and a FOXP3^+^TNFRSF9^-^ Treg cell cluster (**Figure 5A and Figure S7A**). The FOXP3^+^TNFRSF9^-^ Treg cell cluster exhibiting a lower Treg signature score was reported to represent a resting state and might have the potential to gradually transition to the activated state (FOXP3^+^TNFRSF9^+^ Treg) (**Figure 5D**) 34. In the CD8^+^ T cell compartment, we identified eight subsets, including CCR7^+^ Tn, FGFBP2^+^ Teff, IL7R^+^ memory T (Tm), GZMK^+^ Tem, ZNF683^+^ tissue-resident memory T (Trm) and three exhausted T (Tex) cell clusters (**Figure 5A and Figure S7B**). Interestingly, these three Tex cell clusters occupied higher proportions in LP subtype tumors (**Figure 5C**), further validating the immunoinactivated microenvironment in LP subtype tumors. Furthermore, to determine the proliferative ability of T cells, we calculated the proliferation score based on cell cycle genes to denote G1/S or G2/M phases (**Figure S7C-D**). In cycling T cells, we identified both G1/S and G2/M phase clusters of CD8^+^ T cells, CD4^+^ T cells, and Treg cells based on marker expression (**Figure S7D-E**). Intriguingly, we found that cycling CD8^+^ and CD4^+^ T cells highly expressed T cell exhaustion-related genes (**Figure S7F**). These data provide us a comprehensive insight into the tumor-infiltrating T cell landscape in breast cancer and their abundance variation among diverse subtypes.

To gain insight into the composition of NK/ILCs, we reconstructed the clustering of NK/ILCs and identified three main subsets, including two NK cell clusters and a novel ILC3 cluster (**Figure 5E**). FGFBP2^+^ NK cells significantly expressed cytotoxic and effector markers, such as *FGFBP2*, *FCGR3A* and *PRF1*, and revealed enrichments of cytolysis and phagocytosis pathways (**Figure 5F-G**). Additionally, the KLRC1^+^ NK cell subpopulation displayed markers covering *KLRC1*, *XCL1* and *XCL2* and exhibited enrichments of T cell activation and differentiation pathways (**Figure 5D**, **F-G**).

Furthermore, we identified an ILC3 cluster characterized by the expression of *IL7R*, *LTB* (lymphotoxin) and *KIT*, but negative for *KLRG1* (**Figure 5F**) 35. This unusual ILC3 cluster exhibited an enrichment of the leukocyte proliferation pathway (**Figure 5G**). To examine the activity of transcription factors (TFs) among NK/ILCs, we performed pySCENIC to build gene regulatory networks and distinguished different groups of TFs (**Figure S7G**). The activities of a series of regulons including KLF2, TBX21, IKZF1 and XBP1 were found to be elevated in FGFBP2^+^ NK cells, while CEBPB, CEBPD and ELF1 were highly active in KLRC1^+^ NK cells (**Figure S7G**). We also uncovered several regulons incorporating ZNF37A, CEBPG, and FOS underlying the IL7R^+^ ILC3 cluster. Our findings implied that this ILC3 cluster maintained specific transcriptomic properties, which might diversify their functions. Finally, we investigated the prognostic value of this novel IL7R^+^ ILC3 cluster and found that a high composition of the IL7R^+^ ILC3 cluster was strongly linked to longer relapse-free survival (RFS) (**Figure 5H**), suggesting that this novel ILC3 cluster might serve as a robust prognostic biomarker in breast cancer.

Furthermore, we distinguished six distinct B cell clusters, comprising IgM^+^ naïve B (Bn), IgM^-^ Bn, IgM^+^ memory B (Bm), IgM^-^ Bm, ISG15^+^ Bm, and MKI67^+^ cycling B clusters with differentially expressed gene signatures (**Figure S8A**-**B**). For example, IgM^+^ Bn cells featured positive expression of *IGHM* (IgM) and *TCL1A*, a molecule whose expression is restricted to early B cells (**Figure S8B**) 36. The ISG15^+^ Bm cluster occupied a higher proportion in LP subtype tumors, while the MKI67^+^ cycling B cluster was mainly found in mixed tumors (**Figure S8C**). Additionally, plasma cells formed an isolated cluster and revealed significant heterogeneity (**Figure S8D**). Differentially expressed immunoglobin-encoding genes, such as *IGHM*, *IGHG1*, *IGKC* and *IGLC2,* might primarily drive the heterogeneity of plasma cells (**Figure S8E**).

### Myeloid cell heterogeneity in breast cancer

**Figure 6:**
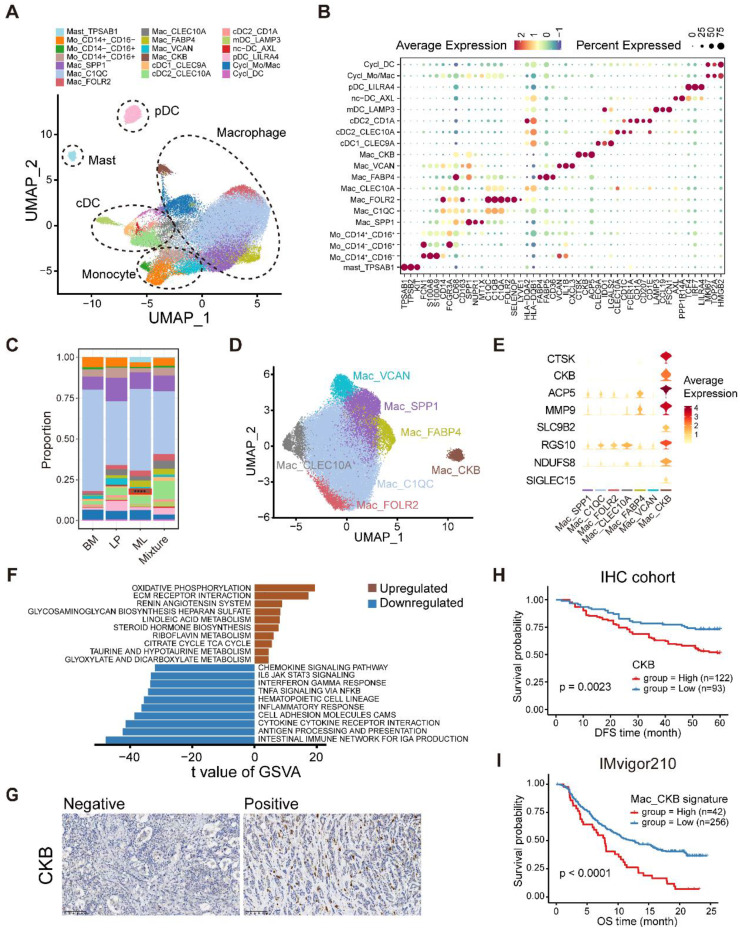
** Myeloid cell heterogeneity in breast cancer.** (A) UMAP plot showing diverse subsets of myeloid cells. (B) Bubble heatmap showing expression levels of selected signature genes in myeloid cells. Dot size indicates fraction of expressing cells, colored based on average normalized expression levels. (C) Bar chart showing the relative proportion of major myeloid cell types in different molecular subtypes. (D) UMAP plot showing seven main subsets of macrophages. (E) Violin plot showing expression levels of selected signature genes in macrophages. (F) Bar plot showing different pathways enriched in CKB^+^ macrophages scored per cell by GSVA. t values are calculated with limma regression. (G) Immunohistochemistry of breast tissue microarray for CKB. (H) Kaplan-Meier plot showing worse clinical outcome in breast cancer patients with higher expression of CKB within our IHC cohort. *P* value is calculated using the log-rank test. (I) Kaplan-Meier plot showing worse clinical outcome in patients receiving anti-PD1 treatment with higher composition of CKB^+^ macrophage subset in the IMvigor210 dataset. *P* value is calculated using the log-rank test.

To further determine the heterogeneity of myeloid cells in the breast tumor microenvironment (TME), we analyzed scRNA-seq data on 52,955 myeloid cells that were unsupervised graph-based clustered into 19 minor subsets based on canonical cell markers (**Figure 6A-B**) 18. The major lineages included mast cells, dendritic cells (DCs), monocytes, macrophages and cycling myeloid cells (**Figure 6A**). Mast cells were characterized by specific high expression of *TPSAB1*, *TPSB2*, and *KIT* and were abundant in ML subtype samples (**Figure 6B-C**). DCs were classified into multiple subtypes based on the expression of classical cell markers, including conventional type 1 and 2 DCs (cDC1s, cDC2s), migratory DCs (mDCs), plasmacytoid DCs (pDCs), and noncanonical DCs (nc-DCs) (**Figure 6A-B and Figure S9A-B**). The CD1A^+^ cDC2 cluster was sparsely present in ML subtype samples and specifically expressed Langerhans cell markers such as *CD1A* and *CD207* (langerin), revealing that this cluster represented langerin^+^ DCs (**Figure 6C and Figure S9B**) 37. We verified strikingly activation and migration signature scores in LAMP3^+^ mDCs, in agreement with the previous study (**Figure S9C**) 18. LAMP3^+^ mDCs had the highest tolerogenic signature score, suggesting a vital role in regulating adaptive responses against peripheral antigens (**Figure S9C and Table S7**) 38. We then identified a unique nc-DC subpopulation expressing markers of both cDCs and pDCs, which might be intermediate between canonical pDCs and cDCs (**Figure S9A**). This subset was defined by several markers, including *AXL* and *SIGLEC6,* consistent with previous research in human blood (**Figure S9B**) 39. Further differential analyses were conducted to explore the differentially expressed genes between this nc-DC subset and cDCs as well as pDCs (**Figure S9D-E**). We revealed that nc-DCs had upregulation of human leukocyte antigen (HLA) genes (*HLA-DPB1* and *HLA-DQB1*) (**Figure S9E**), indicating higher antigen-presenting capacity. Likewise, nc-DCs highly expressed several pDC markers, such as *TCF4* (**Figure S9D**), which is involved in pDC development through a BRD protein-dependent feedback loop 40.

Further clustering of monocytes characterized by *FCN1*, *S100A8*, and *S100A9* enable to generate three subpopulations, covering classical CD14^+^CD16^-^ as well as the nonclassical CD14^+^CD16^+^ and CD14^-^CD16^+^ subgroups (**Figure 6B**, **Figure S10A-B**). Subsequently, macrophages formed seven subpopulations with varying levels of “classically activated” M1-like and “alternatively activated” M2-like macrophage signature scores (**Figure 6D**, **Figure S10C and Table S8**). Among these macrophage subclusters, two TAM subsets, SPP1^+^ TAM and C1QC^+^ TAM, had been reported to exhibit dichotomous functional phenotypes of TAMs (**Figure 6D**) 41. Furthermore, we found a C1QC^+^CLEC10A^+^ TAM subset that might be DC-like TAMs with overexpression of HLA genes (*HLA-DQA2* and *HLA-DQB1*), *CLEC10A* and *CD1E* as well as the higher TAM signature score than those for classical DCs (**Figure 6B, D and Figure S10D**). In addition, we identified a tissue-resident C1QC^+^FOLR2^+^ TAM subset, which was reported to reside in a perivascular niche in the tumor stroma and interacted with tumor-infiltrating CD8^+^ T cells, correlating with a superior prognosis 42. We then found a lipid-associated macrophage (LAM) subpopulation that contributed to an immunosuppressive TME according to our previous study 43. Notably, a novel macrophage subpopulation enriched in ML subtype samples was featured by elevating expression of creatine kinase brain isoform (*CKB*) (**Figure 6C-E and Figure S10E**). CKB is a member of the creatine kinase enzyme family that can reversibly transfer a high-energy phosphate group between ATP and creatine, generating phosphocreatine and ADP 44. Pathway enrichment analysis using GSVA indicated enrichments of oxidative phosphorylation and extracellular matrix (ECM) receptor interaction in this CKB^+^ macrophage subset (**Figure 6F**).

Next, we investigated the sophisticated regulatory network of TFs regulating each macrophage subpopulation via pySCENIC (**Figure S10F**). For example, the LAM subpopulation upregulated the positive regulators MITF and PPARG but downregulated the ATF4 regulon (**Figure S10F**). The results revealed that ELF3, SP6, and ZFY had markedly increasing levels in the regulatory network of CKB^+^ macrophages and might represent the master TFs driving their differentiation (**Figure S10F**). Ultimately, we revealed that higher composition of CKB^+^ macrophages and higher expression of CKB were both linked with a survival disadvantage in the METABRIC cohort (**Figure S10G-H**). In similar, our IHC results further validated that the increase of CKB^+^ cells indicated inferior DFS (**Figure 6G-H**). The composition of CKB^+^ macrophages was positively correlated with immunotherapy resistance and poor outcome in patients receiving ICB treatment (**Figure 6I and Figure S10I**). Taken together, these data illustrate the tumor-infiltrating myeloid cell landscape in breast cancer, and show that the identified novel CKB^+^ macrophage subpopulation may have important value in predicting the prognosis and immunotherapy response in breast cancer.

### The stromal compartment in normal and cancerous breasts comprises diverse cell types

**Figure 7:**
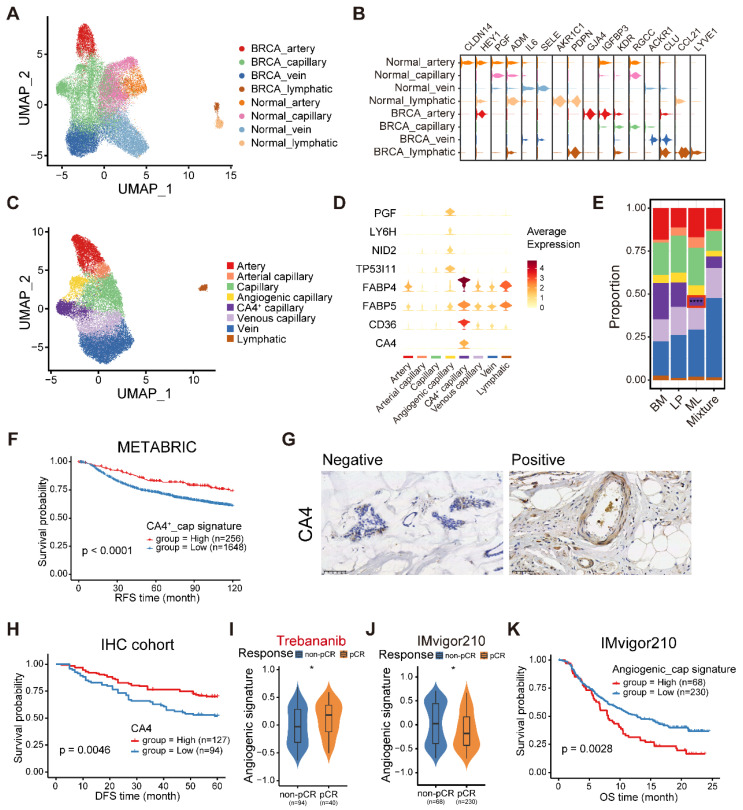
** The endothelial cellular landscape in normal and cancerous breast tissues.** (A) UMAP plot showing diverse subsets of ECs from normal and cancerous breast tissues. (B) Violin plot showing expression levels of selected signature genes in ECs from normal and cancerous breast tissues. (C) UMAP plot showing diverse subsets of breast cancer ECs. (D) Violin plot showing expression levels of selected signature genes in breast cancer ECs. (E) Bar chart showing the relative proportion of major breast cancer EC types in different molecular subtypes. (F) Kaplan-Meier plot showing better clinical outcome in breast cancer patients with higher composition of CA4^+^ capillary subset within the METABRIC cohort. *P* value is calculated using the log-rank test. (G) Immunohistochemistry of breast tissue microarray for CA4. (H) Kaplan-Meier plot showing worse clinical outcome in breast cancer patients with higher expression of CA4 within our IHC cohort. *P* value is calculated using the log-rank test. (I) Violin plot comparing the expression level of angiogenic capillary cell marker genes in breast cancer patients with different responses to antiangiogenic treatment in the I-SPY2 cohort. Unpaired two-sided Wilcoxon test. (J) Violin plot comparing the expression composition of angiogenic capillary subset in breast cancer patients with different responses to anti-PD1 treatment. Unpaired two-sided Wilcoxon test. (K) Kaplan-Meier plot showing worse clinical outcome in patients receiving anti-PD1 treatment with higher composition of angiogenic capillary subset in the IMvigor210 dataset. *P* value is calculated using the log-rank test.

To investigate the heterogeneity of the stromal compartment, including ECs, fibroblasts, and pericytes (also named perivascular-like (PVL) cells), we integrated stromal cells from normal and cancerous breasts and corrected the batch effects. First, we analyzed 33,529 (18,096 from breast cancers, 15,433 from normal breasts) ECs and identified distinct lymphatic vessels and blood vessels of diverse vascular beds (arteries, capillaries, and veins) (**Figure 7A**). We annotated four main endothelial compartments in both normal and cancerous breasts based on the conserved markers (**Figure 7B**) 45. Surprisingly, ECs from normal and cancerous breasts failed to uniformly mix together, revealing obvious biological differences among them. We observed a series of differentially expressed markers in the congeneric endothelial compartment between normal and cancerous breasts. For example, both* PDPN* and *CCL21* were found to overexpress in lymphatic ECs from normal and malignant breasts (**Figure 7B**). Additionally, *AKR1C1* was only discovered in lymphatic ECs from normal breast tissues, while *LYVE1* distinctively expressed in lymphatic ECs from malignancies (**Figure 7B**). To characterize the functions among diverse endothelial compartments, we applied GSVA in hallmark gene sets (**Figure S11A**). Compared with ECs in the normal breasts, the tumor-derived compartments exhibited activation of adipogenesis, angiogenesis and fatty acid metabolism, but downregulation of the reactive oxygen species pathway and mTORC1 signaling (**Figure S11A**). Specifically, multiple pathways including apoptosis, the p53 pathway, and TGF-β signaling, were enriched in the vein compartment of tumors (**Figure S11A**).

In order to further elucidate the heterogeneity of ECs in breast cancer, we first conducted ROGUE analysis. The results determined that capillary ECs exhibited the highest heterogeneity among the four EC subpopulations (**Figure S11B**). Then, the capillary ECs were further re-clustered into five subsets (**Figure 7C**). A subpopulation of capillary ECs expressed both capillary (*RGCC* and *KDR*) and arterial (*GJA4*) markers, named arterial capillary ECs (**Figure S11C**). Analogously, we identified venous capillary ECs cluster in the presence of both capillary (*RGCC* and *KDR*) and venous (*ACKR1*) markers (**Figure S11C**). In addition, a CA4^+^ capillary ECs significantly expressed markers related to uptake and metabolism of fatty acids and glycerol, including *FABP4*, *FABP5* and *CD36* (**Figure 7D and Figure S12A**). An angiogenic capillary EC with higher expression of angiogenic genes (*PGF*) was also defined (**Figure 7D**). We observed variations in the proportions of these capillary EC subtypes, and a markedly decreased proportion of CA4^+^ capillary ECs was found in ML subtype samples (**Figure 7E**). Using GSVA, we assessed the enriched signaling pathways of these two special subsets. Expectedly, the CA4^+^ capillary ECs showed enrichment of the peroxisome proliferator-activated receptor (PPAR) signaling pathway, which is associated with fatty acid sensing and lipid metabolism regulation (**Figure S12B**) 46, whereas the angiogenic capillary ECs exhibited richness in the WNT/β-catenin signaling pathway involved in modulating cell stemness (**Figure S12C**) 47. Next, we examined the regulatory network underlying each capillary EC subset via pySCENIC, and identified specific TF regulons for each capillary EC subset (**Figure S12D**). The CA4^+^ capillary ECs showed increased activities in regulators PPARG and NR1H3 (**Figure S12D**), which play significant roles in lipid homeostasis and lipophagy 48. Specifically, the KLF6 regulon, which is crucial in vascular development, remodeling and response to injury 49, was highly activated in the angiogenic capillary cluster (**Figure S12D**). Furthermore, these two specific capillary ECs subtypes have important clinical implications. The CA4^+^ capillary EC signature was correlated with superior survival in the METABRIC cohort (**Figure 7F**). Additionally, we performed IHC analyses to determine the association between the patient survival and the abundance of CA4^+^ capillary ECs in our cohort, and observed a similar result (**Figure 7G-H**). By contrast, the survival of patients showing higher abundance of angiogenic capillary ECs was significantly shorter (**Figure S12E**). Besides, patients who were sensitive to trebananib, an angiogenesis inhibitor, exhibited elevated angiogenic capillary EC signature scores (**Figure 7I**). In addition, we found that the composition of angiogenic capillary ECs was reduced in patients sensitive to ICB treatment in bladder cancer, and was associated with a poor prognosis (**Figure 7J-K**). Overall, these analyses established a cellular landscape of ECs, and might help elucidate the heterogeneity, function and clinical implication of EC subpopulations in breast cancer. Further investigation is needed to reveal how these special capillary EC clusters influence patient survival and treatment efficacy.

For fibroblasts, by integrating 48,749 fibroblasts from normal breasts and 40,529 cancer-associated fibroblasts (CAFs), we identified nine major cell types (**Figure S13A**). Fibroblasts from normal breasts exhibited low expression of myofibroblast-like CAF (mCAF) markers such as *ACTA2* and *MMP11*, and myogenesis pathway activity deficiency (**Figure S13B-C**). Unexpectedly, we described two subpopulations from normal breasts showing weak expression of *DCN* and *LUM*, two classical markers of fibroblasts (**Figure S13B**). However, they revealed significantly elevated levels of mesenteric estrogen-dependent adipogenesis gene (*MEDAG*) (**Figure S13B**) which contributes to preadipocyte differentiation and lipid accumulation 50, and adipocyte adhesion molecule (*CLMP*) which is critical in adipocyte differentiation and maturation (**Figure S13B**) 51. Thus, we named them adipocyte-associated stromal cells (AASCs). One of AASCs was marked by abundance of glycolysis-associated markers (*ENO1* and *PGK1*) and an enrichment of hypoxia and glycolysis pathways, might representing a stressed state (**Figure S13B-C**). Moreover, CAFs were clustered into five clusters. Among them, a special mCAF cluster with hypoxia features had the highest frequency in BM subtype (**Figure S13D-E**). The results also revealed that the hypoxia mCAF signature was related to shorter survival (**Figure S13F**), demonstrating that this hypoxia mCAF subset might act as a potential predictor for breast cancer patient prognosis.

Finally, 10,278 pericytes from normal breast tissues and 10,489 pericytes from breast cancer tissues were integrated and clustered into two states compatible with an immature and a differentiated phenotype (**Figure S14A**). Immature pericytes (imPVLs) exhibited elevated expression of genes associated with stem cells and adhesion molecules (*PDGFRB*, *CD44*, and *RGS5*) (**Figure S14B**). By comparison, differentiated pericytes (dPVLs) revealed enriched contractile-related genes (*ACTA2* and *TAGLN*) (**Figure S14B**). Although imPVL and dPVL cells were both identified in normal breasts and breast cancers, they were equipped with the substantial difference. For example, *MYH11* was only found in dPVL cells from malignancies but not normal breasts (**Figure S14B**). Functionally, imPVL cells significantly expressed genes involved in cell adhesion and ECM receptor interaction pathways (**Figure S14C**). By contrast, dPVL cells exhibited a mCAF-like signature, including enrichments of vascular smooth muscle contraction and myogenesis pathways (**Figure S14C**). The differences in pericytes between normal and cancerous breasts may underscore the potential role of pericytes in reshaping the TME during tumorigenesis.

## Discussion

The present analysis of comparable normal and cancerous breasts from 216 samples provided a large-scale integrated scRNA-seq profile of breast cancer. Through contrasting malignant cells against reference normal mammary epithelial cell populations, we further unveiled diverse novel molecular subtypes of breast cancer, indicating distinct cell-of-origin patterns. Notably, we identified the LP subtype of breast cancer, which demonstrated a remarkably inferior prognosis. We also screened out and validated PLK1 as a pivotal factor involved in chromosomal instability within LP subtype breast cancer. Our findings suggested that PLK1 could serve as a potential therapeutic target for LP subtype breast cancer. In addition, we described the stromal and immune cell subset abundances in these subtypes and identified novel subpopulations of ILCs, macrophages and endothelial cells associated with tumor progression. Ultimately, the associations between tumors with specific clusters and prognosis were investigated in large-scale bulk transcriptome dataset and validated in our cohort by IHC staining. We unraveled tumor heterogeneity from an evolutive perspective and defined distinct molecular subtypes coupled with the intrinsic capacity of tumors.

Initially, we identified three major epithelial cell subpopulations, including the BM, LP and ML subsets. A recent research generated a multi-dimensional atlas of normal breast tissues and organoids by scRNA-seq and mass cytometry, identified diverse mammary epithelial cell subpopulations similar to those in our study, albeit with different names 52. Furthermore, through evaluating the homology of signature gene expression patterns between normal and malignant epithelial cells, we presented different neoplastic subclusters.

Using deconvolution, the large transcriptome cohort of breast cancer was classified into different malignant subtypes based on cancer cell-of-origin patterns. Among them, the LP-derived tumors mainly comprise basal-like and normal-like types defined in PAM50 53. Similarly, previous studies have demonstrated that *BRCA1*-mutant basal-like breast tumors stem from LP cells but not basal stem/progenitor cells 7,8,54. Recently, Joyce et al. identified an aberrant ERBB3^lo^ LP subpopulation in *BRCA2* mutation breast cancer, and they found ERBB3^lo^ progenitors could give rise to both ER^+^ and ER^-^ cells, potentially acting as the cellular origins for both ER-positive and triple-negative cancers 55. Hence, distinct LP subpopulations could serve as cell-of-origin of different breast cancer subtypes, and this direction need further elaborate exploration. Compared with other subtypes, LP-dominated patients displayed an inferior prognosis but were sensitive to NAC, PARPi and ICB. The correlation between LP-originated cancer cells and genomic alteration profiles was investigated in detail. The intrinsic features of the LP subtype showed high TMB and HRD scores. The LP-high samples also exhibited high levels of aneuploidy and chromosomal instability, while the extrinsic features exhibited the immunosuppresive TME. Subsequently, many immune checkpoints, including *PDCD1*, *HAVCR2*, *LAG3* and *TIGIT,* highly expressed in LP subtype cases, potentially explaining the immunosuppressive microenvironment and high sensitivity to immunotherapy in the LP subtype breast cancer. Finally, conventional biomarkers such as ERα, PR, HER2 and Ki67, fail to distinguish BM type and LP type in clinical practice. Our analysis proposed a novel predictor PAST1 that was the optimal biomarker for LP subtype. Therefore, the diagnostic criteria containing these characteristic biomarkers in breast cancer should be extensively performed in the future.

Through conducting multi-omics analyses including scRNA-seq, bulk RNA-seq and Perturb-seq analyses and both *in vivo* and *in vitro* experiments, our data revealed that PLK1 acted as a major player involved with chromosomal instability within LP subtype breast cancer. Within the domain of LP subtype breast cancers, PLK1 could function as a potential treatment target warranting further exploration. A recent research reported a molecular subtype-dependent prognostic role of PLK1 in breast cancer 56. High PLK1 protein expression was remarkably related to a superior prognosis in the whole breast cancer cohort as well as the luminal subtype, but linked with a poor outcome in the triple-negative breast cancer (TNBC) subtype. Our data found the anti-cancer manifestation upon inhibiting PLK1 in the LP-like cell line and mice tumor model. Regarding the role of PKL1 during treatments, a previous study showed that PLK1 inhibition could overcome therapeutic resistance to bromo- and extraterminal domains (BET) inhibitors in TNBC 57. Besides, in hormone receptor-positive/HER2-negative metastatic breast cancer, high PLK1 mRNA level was found in patients refractory to palbociclib combined with endocrine therapy, indicating PLK1 might be important in the setting of resistance to cyclin-dependent kinase 4/6 (CDK4/6) inhibitors 58. Thus, the role of PLK1 in breast cancer is distinct within each subtype. Further exploration should focus on the molecular mechinism of PLK1-involved chromosome instability within the LP subtype breast cancer.

Interestingly, we described a specific population of IL7R^+^ ILC3 cluster. This cluster is endowed with cytotoxic and naïve traits and upregulates chemokines (*XCL1* and *XCL2*) and lymphotoxins (*LTB*). *XCL1* and *XCL2* recruit cDC1 into the tumor microenvironment to improve tumor immune control 59. In addition, LTB enables increased T cell effector functions and resistance to exhaustion via combination with its receptor LTBR 60. These studies are consistent with our results, in which IL7R^+^ ILC3 cluster functions as a pivotal driver to stimulate leukocyte proliferation, chemotaxis and activation. Some key TFs, such as ZNF37A, CEBPG and FOS, prominently expressed in IL7R^+^ ILC3 and may be involved in their differentiation and activation. Clinically, higher infiltration of IL7R^+^ ILC3 subpopulation is associated with prolonged survival and can be an effective predictor.

Unraveling the diversity of myeloid cells, we identified CKB^+^ macrophages that exist in breast cancer tissues and predict a poor prognosis. This subpopulation specifically expressed metabolic enzymes and transporters (*CKB*, *SLC9B2*, *NDUFS8*) and osteoclast markers (*CTSK*, *ACP5*, *MMP9*, *SIGLEC15*). CKB is an enzyme involved in high-energy phosphoryl transfer between ATP and various phosphogens, such as creatine phosphate 61. Notably, CKB^+^ macrophages showed metabolic activation, including oxidative phosphorylation, lipid metabolism and glycosaminoglycan biosynthesis. However, the origin and development of CKB^+^ macrophages remain unknown. ELF3 is an ETS family transcription factor that mediates macrophage differentiation 62, secretion of inflammatory cytokines in macrophages 63,64, and macrophage proliferation 65. Thus, ELF3 may contribute to CKB^+^ macrophage development. Finally, CKB is a biomarker for novel macrophage subpopulation in breast cancer. Therefore, the biological function and immunotherapy potency of CKB^+^ macrophages in various cancer types should be elaborated in subsequent studies.

By applying clustering approaches, we constructed a blueprint of EC heterogeneity. Briefly, EC types were characterized by functional differences and specific markers within the TME. For example, the subtype of ECs named angiogenic capillaries predicted poor survival, while patients enriched in CA4^+^ ECs had favorable survival. Regarding therapeutic regimens, cases in abundance of angiogenic capillaries were sensitive to antiangiogenic therapy but resistant to ICB treatment. This finding suggests that angiogenic capillaries should be viewed as a predictor of antiangiogenic remedies. Considering the heterogeneity of ECs, they mediate metabolic homeostasis and inflammation depending on diverse subtypes 66. Consistently, CA4^+^ ECs in the TME showed abundance in fatty acid metabolism and oxidation potentially by upregulation of PPARγ 25. During pySCENIC analysis, CA4^+^ EC subpulation was enriched in STAT1 and STAT2, which are involved in interferon and chemokine signaling. This finding suggests that this EC subpopulation can recruit lymphocytes and initiate tumor immune control. Therefore, the versatility of ECs must be considered to refine our understanding of health and malignant diseases.

However, this study has several limitations. First, considering droplet encapsulation and tissue dissociation, certain cell types including granulocytes and adipocytes could not be acquired. Second, the number of cases per clinical subtype was limited. Estimating subtype-specific features will require recruitment of a large, prospective cohort of patients and integration with transcriptional, epigenomic, and clinical readouts.

## Conclusion

In conclusion, the presented single-cell atlas elucidates the cellular repertoire and the cell-of-origin of breast cancer. Our work combining single-cell and bulk RNA-seq data has revealed the evolution mimicry from normal to malignant subtypes and expounded the LP subtype with important clinical implications. Altogether, our data expand the understanding of the complex breast cancer ecosystem and highlight insights into developing novel subtype-specific therapeutic targets for anticancer therapy.

## Supplementary Material

Supplementary figures.

Supplementary tables.

## Abbreviations

BM: basal/myoepithelial
LP: luminal progenitor
ML: mature luminal
scRNA-seq: single-cell RNA sequencing
ILC3: innate lymphoid cell 3
CNV: copy number variant
TMB: tumor mutation burden
HRD: homologous recombination defect
TME: tumor microenvironment
CAFs: cancer-associated fibroblasts
