# Supplementary material for: Single-cell analyses reveal evolution mimicry during the specification of breast cancer subtype
Source: Theranostics. 2024 May 19;14(8):3104–26. doi: 10.7150/thno.96163 (PMC11155410; doi:10.7150/thno.96163)
Supplement: Supplementary file 1 — Supplementary figures. [file thnov14p3104s1.pdf]

Figure S1. Identification of diverse cell types in normal and cancerous breasts by scRNA-seq. Related to Figure 1.

(A) UMAP plots of canonical markers of major cell types in normal breast tissues.

(B) UMAP plots of canonical markers of major cell types in breast cancers.

Figure S2. Delineation of epithelial cells in normal and cancerous breasts. Related to Figure 2.

(A) Bubble heatmap showing expression levels of selected signature genes in normal mammary epithelial cells. Dot size indicates fraction of expressing cells, colored based on average normalized expression levels.

(B) UMAP plot showing 115 minor subsets of 113,254 breast cancer cells.

(C) PCA analysis of the common signature genes among normal epithelial cells and breast cancer cells showing distinct variance from three major cell lineages.

(D) Stacked bar plot showing the composition of three cell lineages in 153 breast tumors containing epithelial cells enrolled in this study. Colors of the bars denote three cell lineages as shown in the legend. The y axis stands for the proportion of each cell lineage in a given tumor sample. Within the x axis, each column represents one tumor case. The annotation bar above denotes the molecular subtypes of tumors that are defined by the dominant cell lineage within each tumor, where yellow represents the mixture of multiple cell lineages.

(E) Consensus clustering of BM-derived cancer cells showing four robust cancer cell subpopulations.

(F) Consensus clustering of LP-derived cancer cells showing eight robust cancer cell subpopulations.

(G) Consensus clustering of ML-derived cancer cells showing eight robust cancer cell subpopulations.

Figure S3. Deconvolution of bulk breast cancer cohort using single-cell signatures.

Related to Figure 3.

(A) Scatterplot showing the proportions of three major lineages. The y axis represents the proportion of LP cells, while the x axis denotes the BM cells proportion, colored based on the proportion of ML cells.

(B) Forest plot showing a multivariate Cox regression analysis for the indicated risk factors in METABRIC cohort.

(C) Violin plot comparing the level of LP proportion in young and aged breast cancer patients within METABRIC cohort. Unpaired two-sided Wilcoxon test.

(D) Violin plot comparing the level of LP proportion in breast cancer patients harboring high and low tumor grade within METABRIC cohort. Unpaired two-sided Wilcoxon test.

(E) Violin plot comparing the level of LP proportion in breast cancer patients harboring positive and negative LNM within METABRIC cohort. Unpaired two-sided Wilcoxon test.

(F) Violin plot comparing the level of LP proportion in breast cancer patients harboring high and low cell cycle score within METABRIC cohort. Unpaired two-sided Wilcoxon test.

Figure S4. Association between LP score and genomic features of breast tumors in TCGA. Related to Figure 3.

(A) Kaplan-Meier plot showing worse clinical outcome in TCGA breast tumors with higher LP score. *P* value is calculated using the log-rank test.

(B) Violin plot comparing the expression level of CN17 between LP-high and LP-low tumors. Unpaired two-sided Wilcoxon test.

(C) Boxplots illustrate the different expression level of diverse copy number signatures (CX2, CX3 and CX5) between LP-high and LP-low tumors. Unpaired two-sided Wilcoxon test.

(D) Violin plot comparing the expression level of *UNG* between LP-low and LP-high breast tumors in TCGA. Unpaired two-sided Wilcoxon test.

(E) Boxplots comparing the omikli and kataegis SBS13 score between LP-low and LP-high breast tumors in TCGA. Unpaired two-sided Wilcoxon test.

(F) Violin plot comparing the expression level of LP score of breast tumors in TCGA with diploid (WGD×0), tetraploid (WGD×1) and octoploid (WGD×2). Kruskal-Wallis test.

(G) Violin plot comparing the aneuploidy score between LP-low and LP-high breast tumors in TCGA. Unpaired two-sided Wilcoxon test.

(H) Violin plot comparing the subclonal genome fraction score between LP-low and LP-high breast tumors in TCGA. Unpaired two-sided Wilcoxon test.

(I) Boxplots comparing the PARPi7 and TP53 score between LP-low and LP-high breast tumors in TCGA. Unpaired two-sided Wilcoxon test.

(J) Pie plot comparing the pCR rate in LP-high and LP-low patients receiving NAC treatment. Chi-square test.

Figure S5. Association between LP score and immune microenvironment of breast tumors. Related to Figure 3.

(A) Violin plot comparing the TCR and BCR richness as well as SNV and indel neoantigen score between LP-low and LP-high breast tumors in TCGA. Unpaired two-sided Wilcoxon test.

(B) Boxplots illustrate the different immune cell proportions between breast tumors with high and low LP proportion in METABRIC and TCGA cohort. Unpaired two-sided Wilcoxon test.

(C) Boxplots comparing the expression level of a series of immune checkpoint markers between LP-high and LP-low breast tumors in TCGA. Unpaired two-sided Wilcoxon test.

(D) Pie plots comparing the pCR rates in LP-high and LP-low patients receiving paclitaxel and paclitaxel + anti-PD1 treatment. Chi-square test.

(E) Scatterplot showing the correlation with LP score at transcriptional and protein level. Spearman correlation.

(F) Boxplots comparing the expression levels of *PSAT1*, *ER* and *CK14* between diverse molecular subtype breast tumors in METRBRIC cohort. Kruskal-Wallis test.

(G) Kaplan-Meier plot showing worse clinical outcome in our IHC cohort with higher expression of PSAT1. *P* value is calculated using the log-rank test.

(H) Kaplan-Meier plot showing comparable clinical outcome in our IHC cohort with higher expression of ER. *P* value is calculated using the log-rank test.

(I) Kaplan-Meier plot showing worse clinical outcome in our IHC cohort with higher expression of CK14. *P* value is calculated using the log-rank test.

Figure S6. Identification of potential therapeutic targets in the LP subtype breast cancer. Related to Figure 4.

(A) RT-qPCR analyses of PLK1 in PLK1 knockdown (siPLK1) SUM-149PT and MCF-7 cell lines.

(B) RT-qPCR analyses of TPX2 in TPX2 knockdown (siTPX2) SUM-149PT and MCF-7 cell lines.

(C) RT-qPCR analyses of CDK1 in CDK1 knockdown (siCDK1) SUM-149PT and MCF-7 cell lines.

(D) RT-qPCR analyses of AURKA in AURKA knockdown (siAURKA) SUM-149PT and MCF-7 cell lines.

(E) RT-qPCR analyses of PLK1 in PLK1 knockdown (shPLK1) SUM-149PT cells.

Figure S7. Integrated analyses of lymphocytes, NK cells and ILCs. Related to Figure 5.

(A) UMAP plot showing seven $CD4^+$ T cell subpopulations.

(B) UMAP plot showing eight $CD8^+$ T cell subpopulations.

(C) UMAP plot showing the cell cycle score of each cell in the cycling T cluster.

(D) UMAP plot showing diverse states and clusters of cycling T cells.

(E) Bubble heatmap showing expression levels of selected signature genes in cycling T

cells. Dot size indicates fraction of expressing cells, colored based on average normalized expression levels.

(F) UMAP plot showing expression levels of T cell exhaustion marker genes in cycling T cells.

(G) Heatmap showing TF activity for each NK/ILC subset.

Figure S8. Characterization of diverse subsets of B and plasma cells. Related to Figure 5.

(A) UMAP plot showing diverse subsets of B cells.

(B) UMAP plot showing expression levels of selected signature genes in B cells.

(C) Bar chart showing the relative proportion of major B cell type in different molecular subtypes.

(D) UMAP plot showing distinct clusters of plasma cells.

(E) UMAP plot showing expression levels of selected signature genes in plasma cells.

Figure S9. Characterization of diverse subsets of DCs. Related to Figure 5.

(A) UMAP plot showing diverse subsets of DCs.

(B) UMAP plot showing expression levels of selected signature genes in DCs.

(C) Bubble heatmap showing expression levels of the activation, migration, and tolerogenic signatures in DCs. Dot size indicates fraction of expressing cells, colored based on average normalized expression levels.

(D) Volcano plot showing the differentially expressed genes (nc-DC versus pDC cluster). Unpaired two-sided Wilcoxon test.

(E) Volcano plot showing the differentially expressed genes (nc-DC versus cDC cluster). Unpaired two-sided Wilcoxon test.

Figure S10. Characterization of diverse subsets of monocytes and macrophages.

Related to Figure 6.

(A) UMAP plot showing diverse subsets of monocytes.

(B) UMAP plot showing expression levels of *CD14* and *FCGR3A* (CD16) in monocytes.

(C) Heatmap showing different expression patterns of M1 and M2 signature genes among diverse macrophage subsets.

(D) Violin plot comparing the expression level of TAM signature genes between Mac_CLEC10A and cDC2_CLEC10A cluster. Unpaired two-sided Wilcoxon test.

(E) UMAP plot showing expression levels of *CKB* in all lineages of breast cancer.

(F) Heatmap showing TF activity for each macrophage subset.

(G) Kaplan-Meier plot showing worse clinical outcome in breast cancer patients with higher composition of CKB^+ macrophages within the METABRIC cohort. *P* value is calculated using the log-rank test.

(H) Kaplan-Meier plot showing worse clinical outcome in breast cancer patients with higher expression of *CKB* within the METABRIC cohort. *P* value is calculated using the log-rank test.

(I) Violin plot comparing the expression composition of CKB^+ macrophage subset in patients with different responses to ICB treatment in the IMvigor210 dataset. Unpaired two-sided Wilcoxon test.

Figure S11. Characterization of diverse subsets of ECs in normal and cancerous breast tissues. Related to Figure 7.

(A) Heatmap showing enrichment level of hallmark pathways for each endothelial subset from normal and cancerous breast tissues.

(B) Boxplot showing cell purity for breast cancer ECs within four major clusters. Kruskal-Wallis test.

(C) UMAP plot showing expression levels of selected signature genes in breast cancer ECs.

Figure S12. Characterization of novel subsets of ECs in breast cancer tissues. Related to Figure 7.

(A) UMAP plot showing expression levels of *CA4* in all lineages of breast cancer.

(B) Bar plot showing up-regulated pathways enriched in $FABP4^{+}$ capillary cluster scored per cell by GSVA. t values are calculated with limma regression.

(C) Bar plot showing up-regulated pathways enriched in angiogenic capillary cluster scored per cell by GSVA. t values are calculated with limma regression.

(D) Heatmap showing TF activity for each breast cancer EC subset.

(E) Kaplan-Meier plot showing worse clinical outcome in breast cancer patients with higher composition of angiogenic capillary subset within the METABRIC cohort. *P* value is calculated using the log-rank test.

Figure S13. Characterization of diverse subsets of fibroblasts in normal and cancerous breast tissues. Related to Figure 7.

(A) UMAP plot showing diverse subsets of fibroblasts from normal and cancerous breast tissues.

(B) Violin plot showing expression levels of selected signature genes in fibroblasts from normal and cancerous breast tissues.

(C) Heatmap showing enrichment level of hallmark pathways for each fibroblast subset from normal and cancerous breast tissues.

(D) UMAP plot showing diverse subsets of CAFs.

(E) Bar chart showing the relative proportion of major CAF subsets in different molecular subtypes.

(F) Kaplan-Meier plot showing better clinical outcome in breast cancer patients with higher expression of mCAF_ENO1 cell marker genes within the METABRIC cohort. *P* value is calculated using the log-rank test.

Figure S14. Characterization of diverse subsets of pericytes in normal and

cancerous breast tissues. Related to Figure 7.

(A) UMAP plot showing diverse subsets of pericytes from normal and cancerous breast tissues.

(B) Violin plot showing expression levels of selected signature genes in pericytes from normal and cancerous breast tissues.

(C) Bar plot showing different pathways enriched in dPVL and imPVL in breast cancers scored per cell by GSVA. t values are calculated with limma regression.

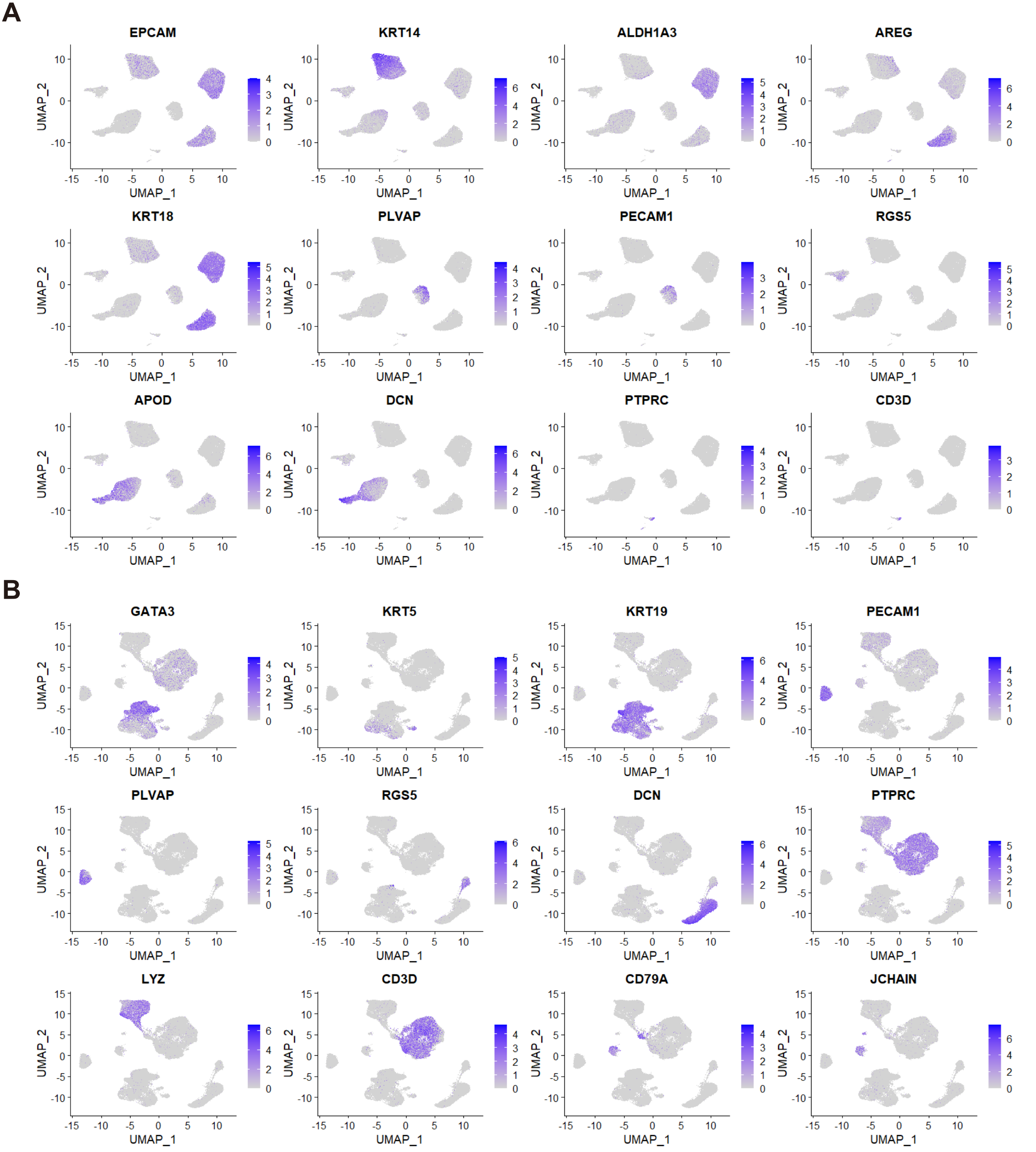
A
EPCAM
KRT14
ALDH1A3
AREG
KRT18
PLVAP
PECAM1
RGS5
APOD
DCN
PTPRC
CD3D
B
GATA3
KRT5
KRT19
PECAM1
PLVAP
RGS5
DCN
PTPRC
LYZ
CD3D
CD79A
JCHAIN
UMAP_1
UMAP_2

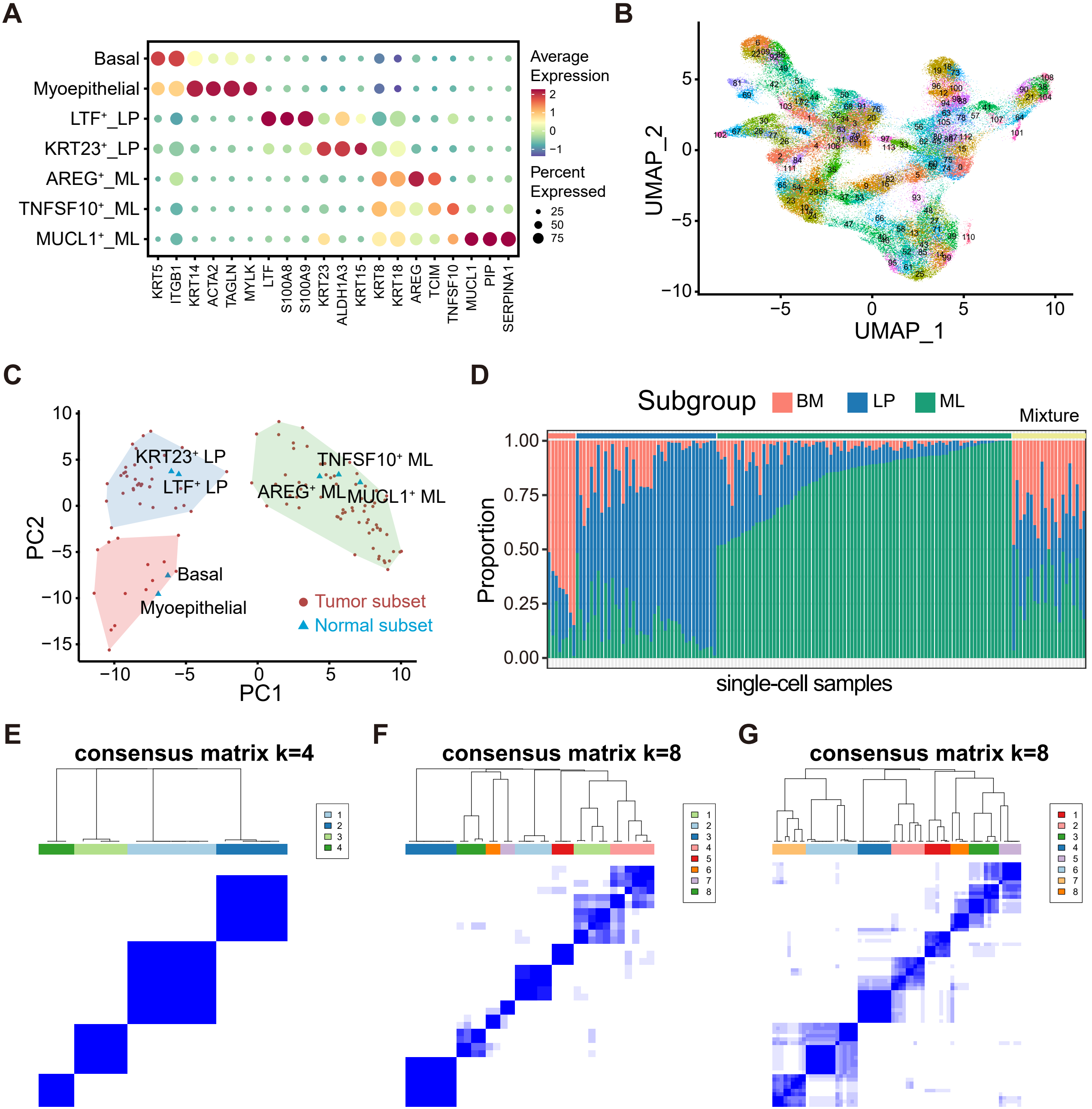

A
Basal
Myoepithelial
LTF⁺_LP
KRT23⁺_LP
AREG⁺_ML
TNFSF10⁺_ML
MUCL1⁺_ML
KRT5
ITGB1
KRT14
ACTA2
TAGLN
MYLK
LTF
S100A8
S100A9
KRT23
ALDH1A3
KRT15
KRT8
KRT18
AREG
TCIM
TNFSF10
MUCL1
PIP
SERPINA1
Average Expression
Percent Expressed
B
UMAP_1
UMAP_2
C
KRT23⁺ LP
LTF⁺ LP
TNFSF10⁺ ML
AREG⁺ ML
MUCL1⁺ ML
Basal
Myoepithelial
Tumor subset
Normal subset
PC1
PC2
D
Subgroup
BM
LP
ML
Mixture
Proportion
single-cell samples
E
consensus matrix k=4
F
consensus matrix k=8
G
consensus matrix k=8

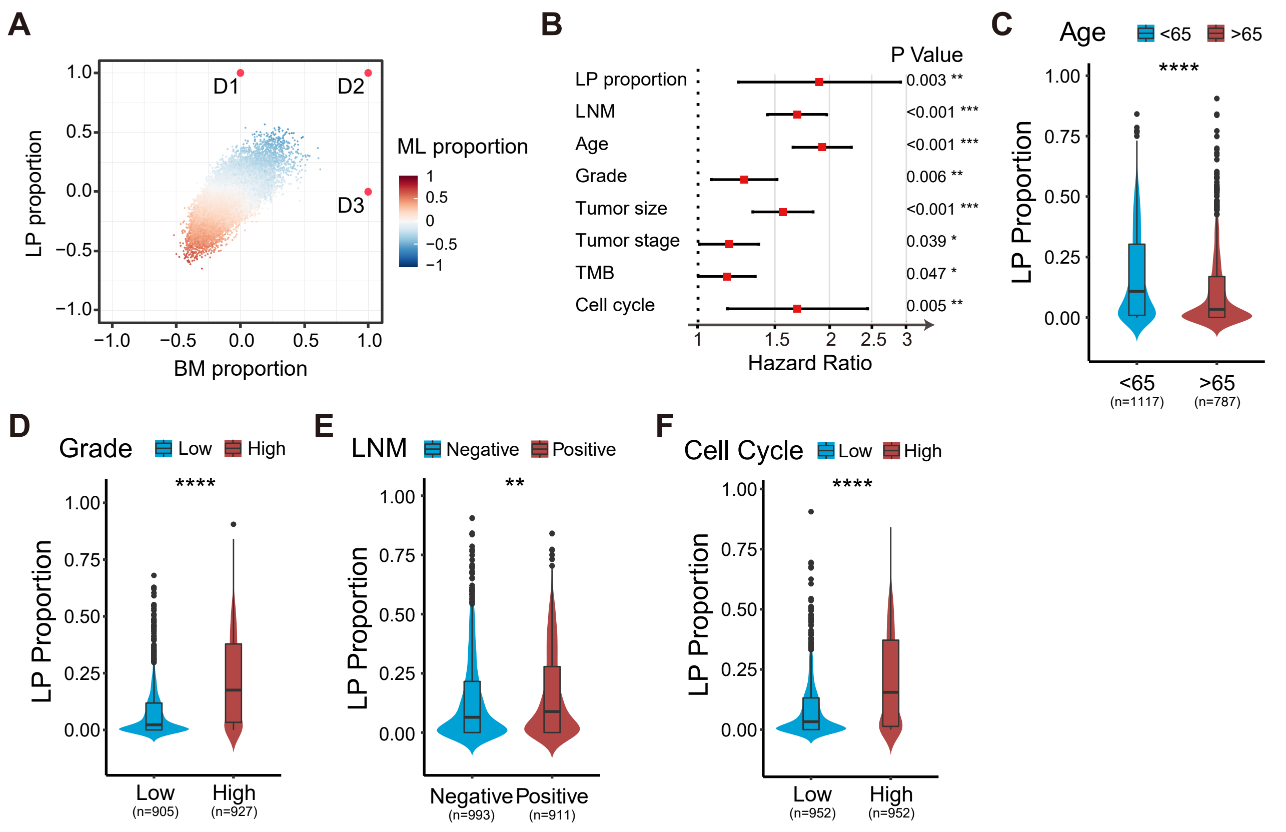

A
LP proportion
BM proportion
D1
D2
D3
ML proportion
1
0.5
0
−0.5
−1
1.0
0.5
0.0
−0.5
−1.0
B
P Value
LP proportion 0.003 **
LNM <0.001 ***
Age <0.001 ***
Grade 0.006 **
Tumor size <0.001 ***
Tumor stage 0.039 *
TMB 0.047 *
Cell cycle 0.005 **
1 1.5 2 2.5 3
Hazard Ratio
C
Age <65 >65
****
LP Proportion
1.00
0.75
0.50
0.25
0.00
<65 (n=1117)
>65 (n=787)
D
Grade Low High
****
LP Proportion
Low (n=905)
High (n=927)
E
LNM Negative Positive
**
LP Proportion
Negative (n=993)
Positive (n=911)
F
Cell Cycle Low High
****
LP Proportion
Low (n=952)
High (n=952)

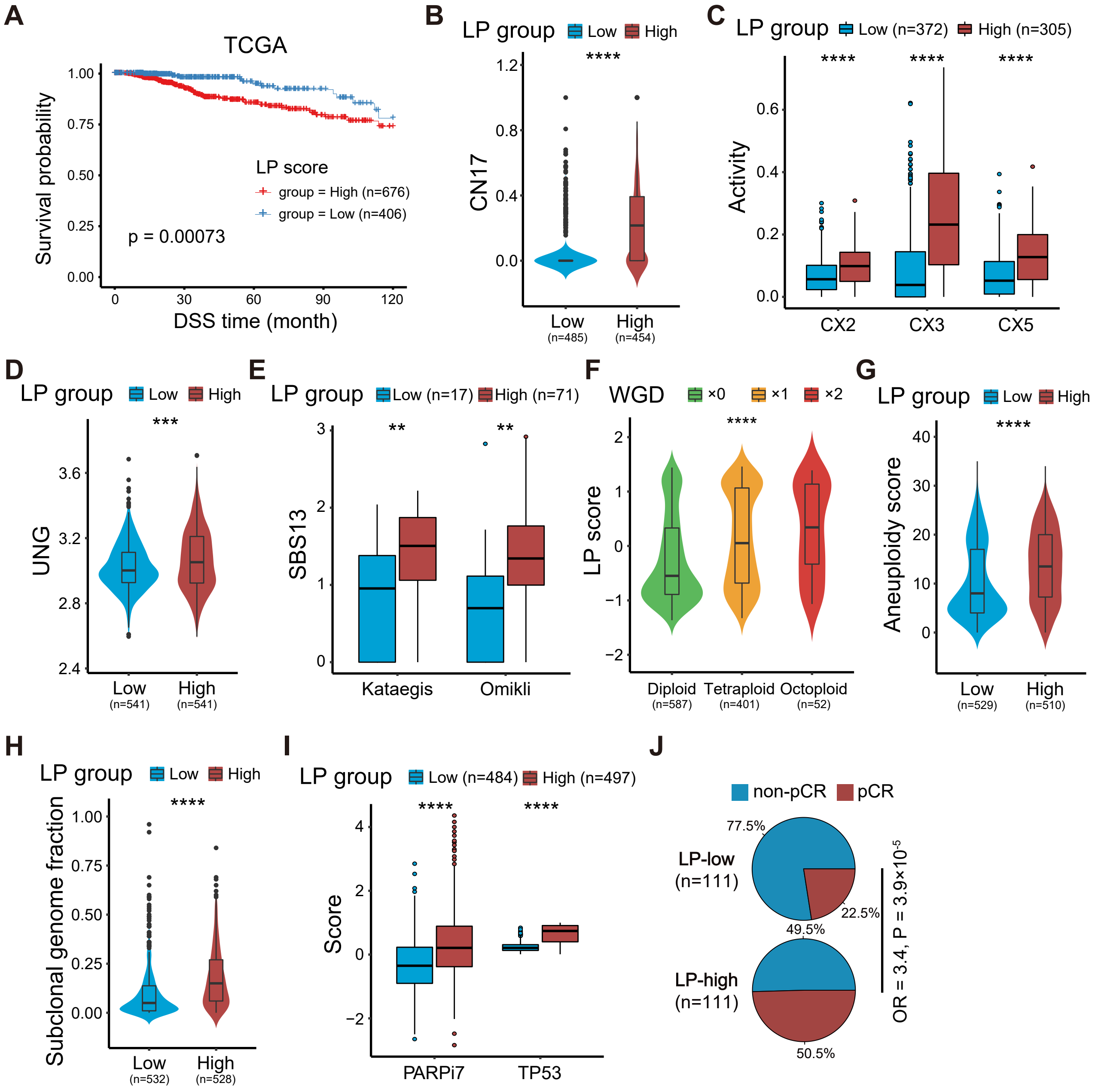
A
TCGA
Survival probability
DSS time (month)
LP score
group = High (n=676)
group = Low (n=406)
p = 0.00073
B
LP group Low High
CN17
Low (n=485)
High (n=454)
C
LP group Low (n=372) High (n=305)
Activity
CX2
CX3
CX5
D
LP group Low High
UNG
Low (n=541)
High (n=541)
E
LP group Low (n=17) High (n=71)
SBS13
Kataegis
Omikli
F
WGD ×0 ×1 ×2
LP score
Diploid (n=587)
Tetraploid (n=401)
Octoploid (n=52)
G
LP group Low High
Aneuploidy score
Low (n=529)
High (n=510)
H
LP group Low High
Subclonal genome fraction
Low (n=532)
High (n=528)
I
LP group Low (n=484) High (n=497)
Score
PARPi7
TP53
J
non-pCR pCR
LP-low (n=111)
77.5%
22.5%
LP-high (n=111)
49.5%
50.5%
OR = 3.4, P = 3.9×10-5

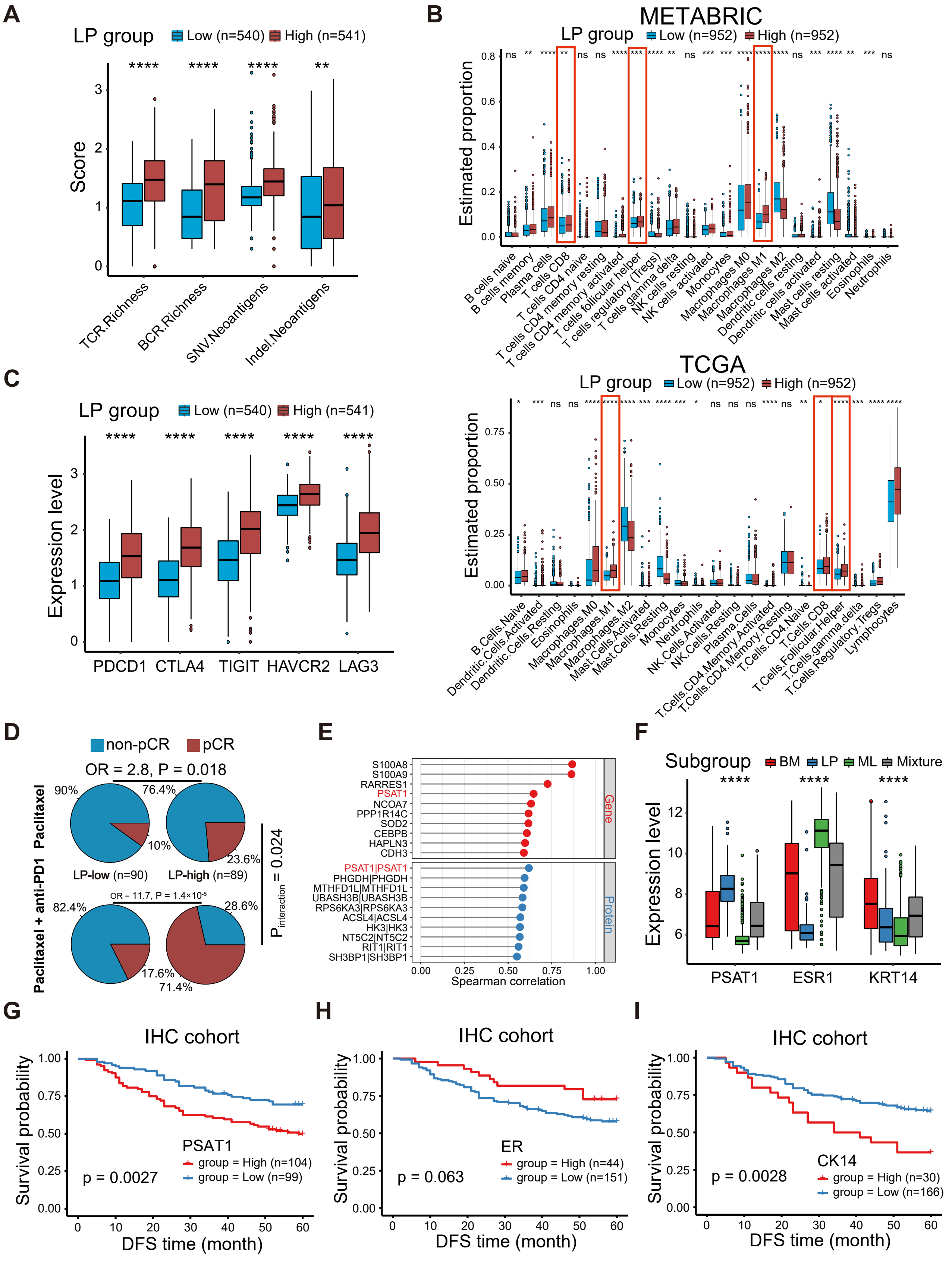
A
LP group Low (n=540) High (n=541)
Score
TCR.Richness
BCR.Richness
SNV.Neoantigens
Indel.Neoantigens
B
METABRIC
LP group Low (n=952) High (n=952)
Estimated proportion
B cells naive
B cells memory
Plasma cells
T cells CD8
T cells CD4 naive
T cells CD4 memory resting
T cells CD4 memory activated
T cells follicular helper
T cells regulatory (Tregs)
T cells gamma delta
NK cells resting
NK cells activated
Monocytes
Macrophages M0
Macrophages M1
Macrophages M2
Dendritic cells resting
Dendritic cells activated
Mast cells resting
Mast cells activated
Eosinophils
Neutrophils
TCGA
LP group Low (n=952) High (n=952)
Estimated proportion
B.Cells.Naive
Dendritic.Cells.Activated
Dendritic.Cells.Resting
Eosinophils
Macrophages.M0
Macrophages.M1
Macrophages.M2
Mast.Cells.Activated
Mast.Cells.Resting
Monocytes
Neutrophils
NK.Cells.Activated
NK.Cells.Resting
Plasma.Cells
T.Cells.CD4.Memory.Activated
T.Cells.CD4.Memory.Resting
T.Cells.CD4.Naive
T.Cells.CD8
T.Cells.Follicular.Helper
T.Cells.gamma.delta
T.Cells.Regulatory.Tregs
Lymphocytes
C
LP group Low (n=540) High (n=541)
Expression level
PDCD1 CTLA4 TIGIT HAVCR2 LAG3
D
non-pCR pCR
OR = 2.8, P = 0.018
Paclitaxel
90% 10% 76.4% 23.6%
LP-low (n=90) LP-high (n=89)
OR = 11.7, P = 1.4×10-5
Paclitaxel + anti-PD1
82.4% 17.6% 28.6% 71.4%
Pinteraction = 0.024
E
S100A8
S100A9
RARRES1
PSAT1
NCOA7
PPP1R14C
SOD2
CEBPB
HAPLN3
CDH3
Gene
PSAT1|PSAT1
PHGDH|PHGDH
MTHFD1L|MTHFD1L
UBASH3B|UBASH3B
RPS6KA3|RPS6KA3
ACSL4|ACSL4
HK3|HK3
NT5C2|NT5C2
RIT1|RIT1
SH3BP1|SH3BP1
Protein
Spearman correlation
F
Subgroup BM LP ML Mixture
Expression level
PSAT1 ESR1 KRT14
G
IHC cohort
Survival probability
PSAT1
group = High (n=104)
group = Low (n=99)
p = 0.0027
DFS time (month)
H
IHC cohort
Survival probability
ER
group = High (n=44)
group = Low (n=151)
p = 0.063
DFS time (month)
I
IHC cohort
Survival probability
CK14
group = High (n=30)
group = Low (n=166)
p = 0.0028
DFS time (month)

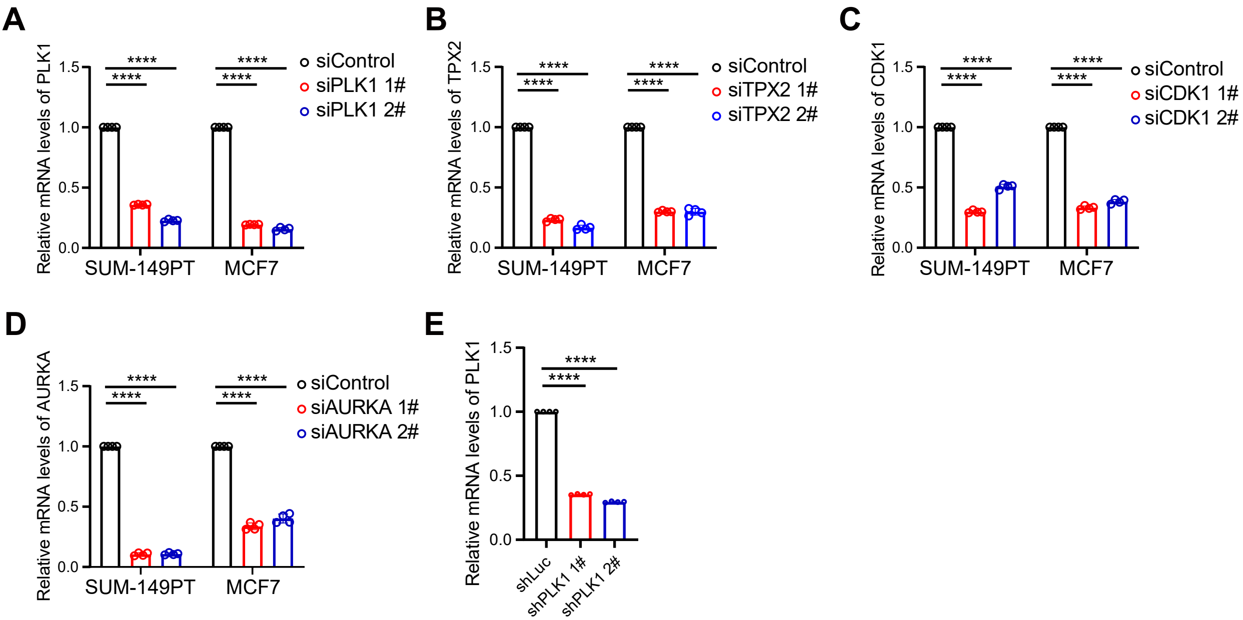
A
Relative mRNA levels of PLK1
1.5
1.0
0.5
0.0
****
****
****
****
SUM-149PT
MCF7
siControl
siPLK1 1#
siPLK1 2#
B
Relative mRNA levels of TPX2
1.5
1.0
0.5
0.0
****
****
****
****
SUM-149PT
MCF7
siControl
siTPX2 1#
siTPX2 2#
C
Relative mRNA levels of CDK1
1.5
1.0
0.5
0.0
****
****
****
****
SUM-149PT
MCF7
siControl
siCDK1 1#
siCDK1 2#
D
Relative mRNA levels of AURKA
1.5
1.0
0.5
0.0
****
****
****
****
SUM-149PT
MCF7
siControl
siAURKA 1#
siAURKA 2#
E
Relative mRNA levels of PLK1
1.5
1.0
0.5
0.0
****
****
shLuc
shPLK1 1#
shPLK1 2#

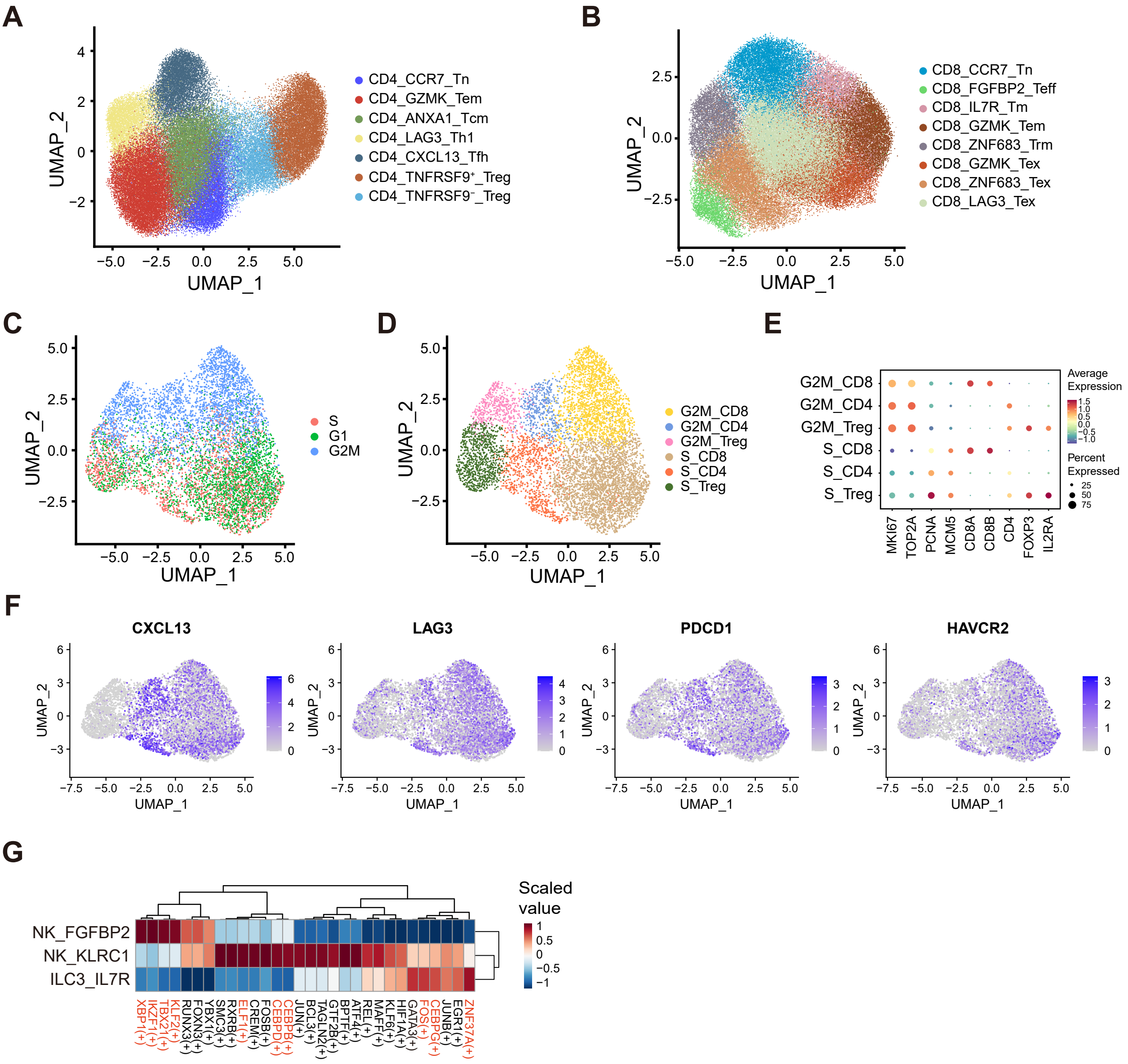
A
B
C
D
E
F
G
UMAP_1
UMAP_2
CD4_CCR7_Tn
CD4_GZMK_Tem
CD4_ANXA1_Tcm
CD4_LAG3_Th1
CD4_CXCL13_Tfh
CD4_TNFRSF9⁺_Treg
CD4_TNFRSF9⁻_Treg
CD8_CCR7_Tn
CD8_FGFBP2_Teff
CD8_IL7R_Tm
CD8_GZMK_Tem
CD8_ZNF683_Trm
CD8_GZMK_Tex
CD8_ZNF683_Tex
CD8_LAG3_Tex
S
G1
G2M
G2M_CD8
G2M_CD4
G2M_Treg
S_CD8
S_CD4
S_Treg
Average Expression
Percent Expressed
MKI67
TOP2A
PCNA
MCM5
CD8A
CD8B
CD4
FOXP3
IL2RA
CXCL13
LAG3
PDCD1
HAVCR2
Scaled value
NK_FGFBP2
NK_KLRC1
ILC3_IL7R
XBP1(+)
IKZF1(+)
TBX21(+)
KLF2(+)
RUNX3(+)
FOXN3(+)
YBX1(+)
SMC3(+)
RXRB(+)
ELF1(+)
CREM(+)
FOSB(+)
CEBPD(+)
CEBPB(+)
JUN(+)
BCL3(+)
TAGLN2(+)
GTF2B(+)
BPTF(+)
ATF4(+)
REL(+)
MAFF(+)
KLF6(+)
HIF1A(+)
GATA3(+)
FOS(+)
CEBPG(+)
JUNB(+)
EGR1(+)
ZNF37A(+)

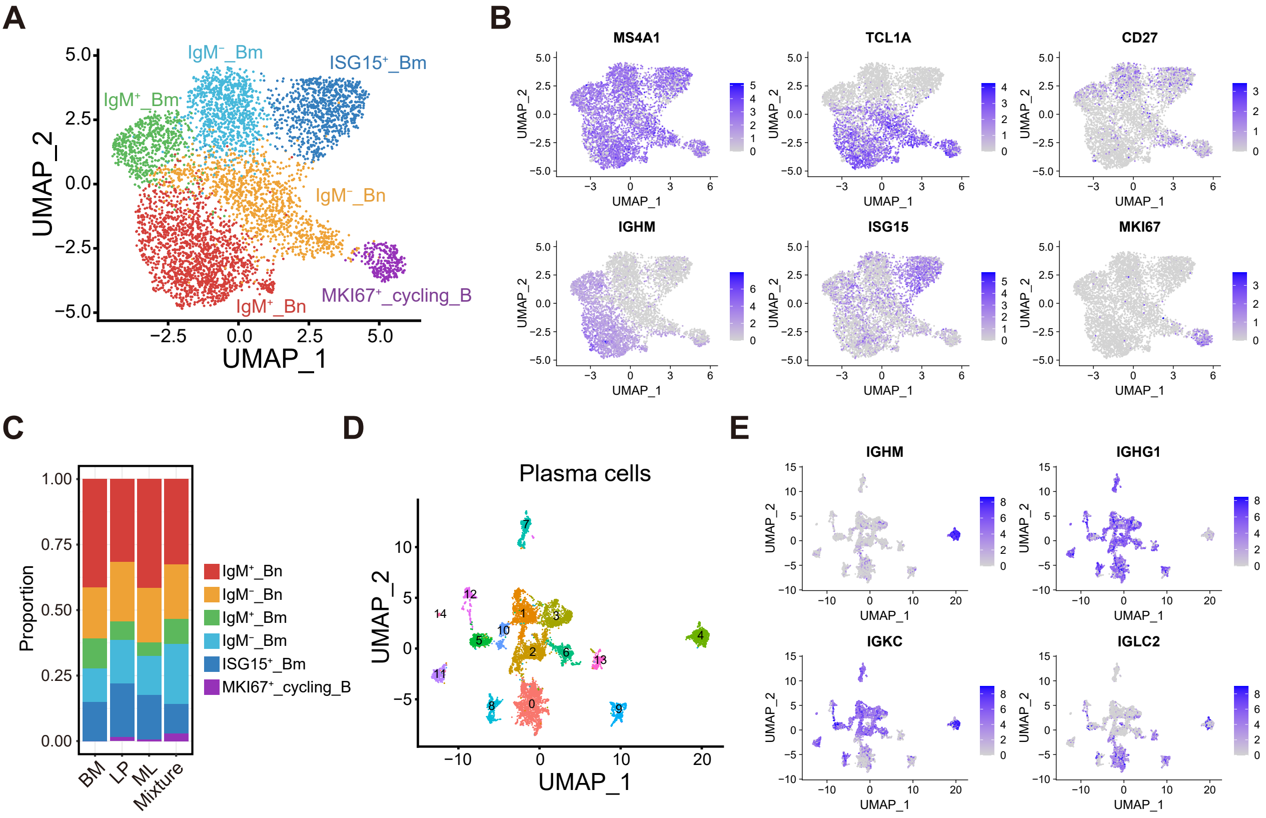

A
IgM⁻_Bm
ISG15⁺_Bm
IgM⁺_Bm
IgM⁻_Bn
IgM⁺_Bn
MKI67⁺_cycling_B
UMAP_2
UMAP_1
B
MS4A1
TCL1A
CD27
IGHM
ISG15
MKI67
C
Proportion
IgM⁺_Bn
IgM⁻_Bn
IgM⁺_Bm
IgM⁻_Bm
ISG15⁺_Bm
MKI67⁺_cycling_B
BM
LP
ML
Mixture
D
Plasma cells
E
IGHM
IGHG1
IGKC
IGLC2

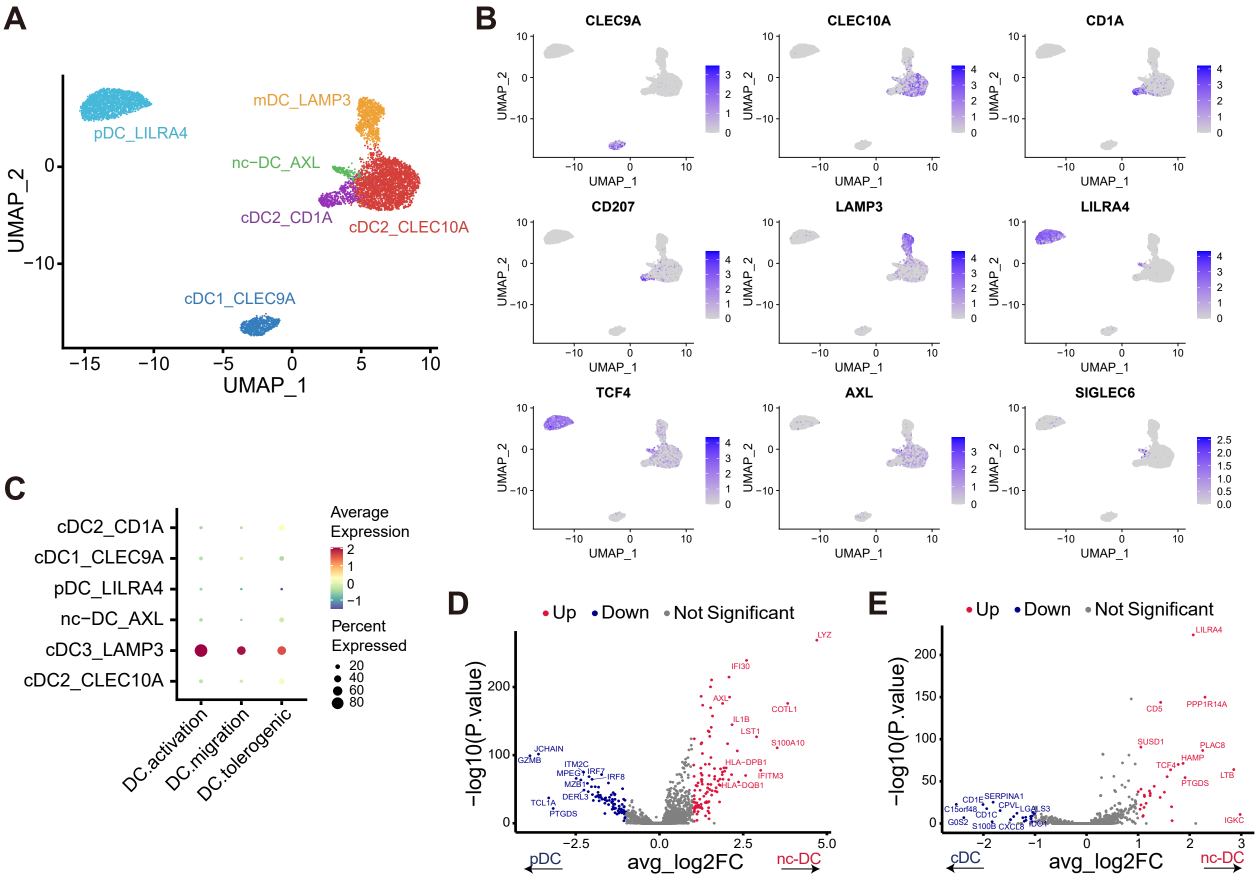

A
pDC_LILRA4
mDC_LAMP3
nc−DC_AXL
cDC2_CD1A
cDC2_CLEC10A
cDC1_CLEC9A
UMAP_1
UMAP_2
B
CLEC9A
CLEC10A
CD1A
CD207
LAMP3
LILRA4
TCF4
AXL
SIGLEC6
C
cDC2_CD1A
cDC1_CLEC9A
pDC_LILRA4
nc−DC_AXL
cDC3_LAMP3
cDC2_CLEC10A
DC.activation
DC.migration
DC.tolerogenic
Average Expression
Percent Expressed
D
Up
Down
Not Significant
-log10(P.value)
avg_log2FC
pDC
nc-DC
LYZ
IFI30
AXL
COTL1
IL1B
LST1
S100A10
HLA-DPB1
IFITM3
HLA-DQB1
JCHAIN
GZMB
ITM2C
MPEG1
IRF7
IRF8
MZB1
DERL3
TCL1A
PTGDS
E
Up
Down
Not Significant
-log10(P.value)
avg_log2FC
cDC
nc-DC
LILRA4
CD5
PPP1R14A
SUSD1
PLAC8
HAMP
TCF4
PTGDS
LTB
IGKC
CD1E
SERPINA1
C15orf48
CPVL
CD1C
LGALS3
G0S2
S100B
CXCL8
IDO1

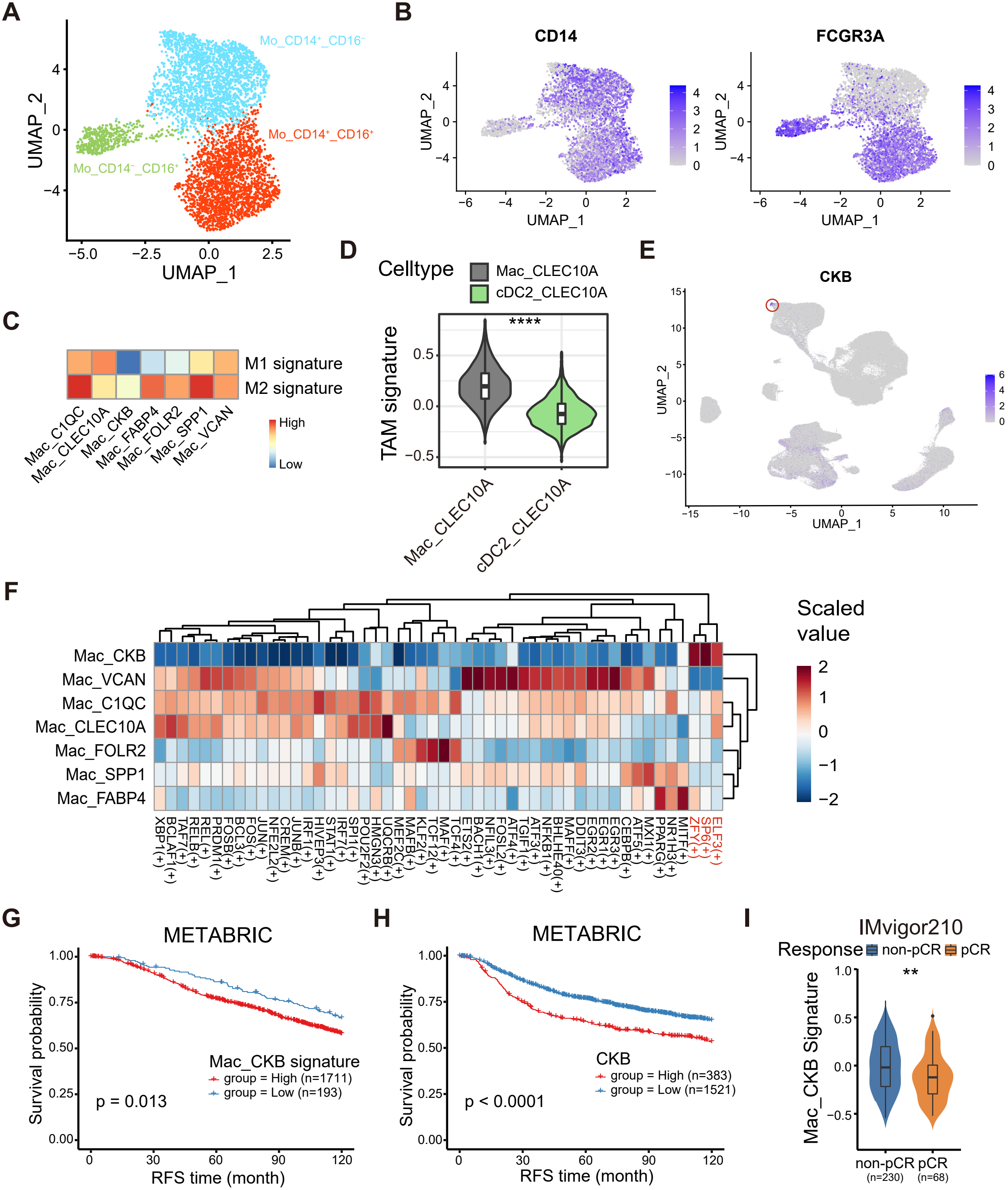

A
Mo_CD14⁺_CD16⁻
Mo_CD14⁺_CD16⁺
Mo_CD14⁻_CD16⁺
UMAP_2
UMAP_1
B
CD14
FCGR3A
UMAP_2
UMAP_1
C
M1 signature
M2 signature
Mac_C1QC
Mac_CLEC10A
Mac_CKB
Mac_FABP4
Mac_FOLR2
Mac_SPP1
Mac_VCAN
High
Low
D
Celltype
Mac_CLEC10A
cDC2_CLEC10A
TAM signature
****
E
CKB
UMAP_2
UMAP_1
F
Scaled value
Mac_CKB
Mac_VCAN
Mac_C1QC
Mac_CLEC10A
Mac_FOLR2
Mac_SPP1
Mac_FABP4
XBP1(+) BCLAF1(+) TAF7(+) RELB(+) REL(+) PRDM1(+) FOSB(+) BCL3(+) FOS(+) JUN(+) NFE2L2(+) CREM(+) JUNB(+) IRF1(+) HIVEP3(+) STAT1(+) IRF7(+) SPI1(+) POU2F2(+) HMGN3(+) UQCRB(+) MEF2C(+) MAFB(+) KLF2(+) TCF12(+) MAF(+) TCF4(+) ETS2(+) BACH1(+) NFIL3(+) FOSL2(+) ATF4(+) TGIF1(+) ATF3(+) NFKB1(+) BHLHE40(+) MAFF(+) DDIT3(+) EGR2(+) EGR1(+) EGR3(+) CEBPB(+) ATF5(+) MXI1(+) PPARG(+) NR1H3(+) MITF(+) ZFY(+) SP6(+) ELF3(+)
G
METABRIC
Survival probability
RFS time (month)
Mac_CKB signature
group = High (n=1711)
group = Low (n=193)
p = 0.013
H
METABRIC
Survival probability
RFS time (month)
CKB
group = High (n=383)
group = Low (n=1521)
p < 0.0001
I
IMvigor210
Response non-pCR pCR
Mac_CKB Signature
**
non-pCR (n=230)
pCR (n=68)

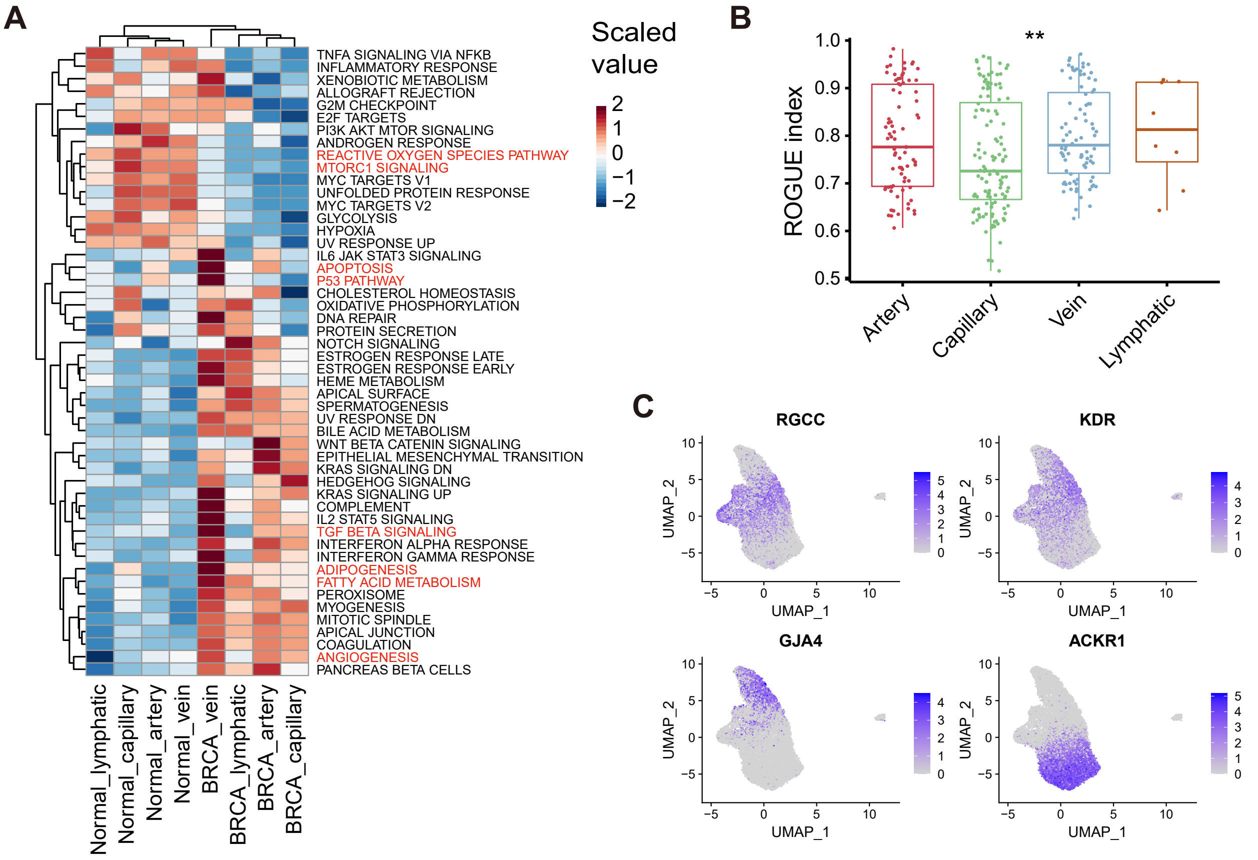
A
Scaled value
2
1
0
−1
−2
TNFA SIGNALING VIA NFKB
INFLAMMATORY RESPONSE
XENOBIOTIC METABOLISM
ALLOGRAFT REJECTION
G2M CHECKPOINT
E2F TARGETS
PI3K AKT MTOR SIGNALING
ANDROGEN RESPONSE
REACTIVE OXYGEN SPECIES PATHWAY
MTORC1 SIGNALING
MYC TARGETS V1
UNFOLDED PROTEIN RESPONSE
MYC TARGETS V2
GLYCOLYSIS
HYPOXIA
UV RESPONSE UP
IL6 JAK STAT3 SIGNALING
APOPTOSIS
P53 PATHWAY
CHOLESTEROL HOMEOSTASIS
OXIDATIVE PHOSPHORYLATION
DNA REPAIR
PROTEIN SECRETION
NOTCH SIGNALING
ESTROGEN RESPONSE LATE
ESTROGEN RESPONSE EARLY
HEME METABOLISM
APICAL SURFACE
SPERMATOGENESIS
UV RESPONSE DN
BILE ACID METABOLISM
WNT BETA CATENIN SIGNALING
EPITHELIAL MESENCHYMAL TRANSITION
KRAS SIGNALING DN
HEDGEHOG SIGNALING
KRAS SIGNALING UP
COMPLEMENT
IL2 STAT5 SIGNALING
TGF BETA SIGNALING
INTERFERON ALPHA RESPONSE
INTERFERON GAMMA RESPONSE
ADIPOGENESIS
FATTY ACID METABOLISM
PEROXISOME
MYOGENESIS
MITOTIC SPINDLE
APICAL JUNCTION
COAGULATION
ANGIOGENESIS
PANCREAS BETA CELLS
Normal_lymphatic
Normal_capillary
Normal_artery
Normal_vein
BRCA_vein
BRCA_lymphatic
BRCA_artery
BRCA_capillary
B
ROGUE index
1.0
0.9
0.8
0.7
0.6
0.5
**
Artery
Capillary
Vein
Lymphatic
C
RGCC
KDR
GJA4
ACKR1
UMAP_1
UMAP_2

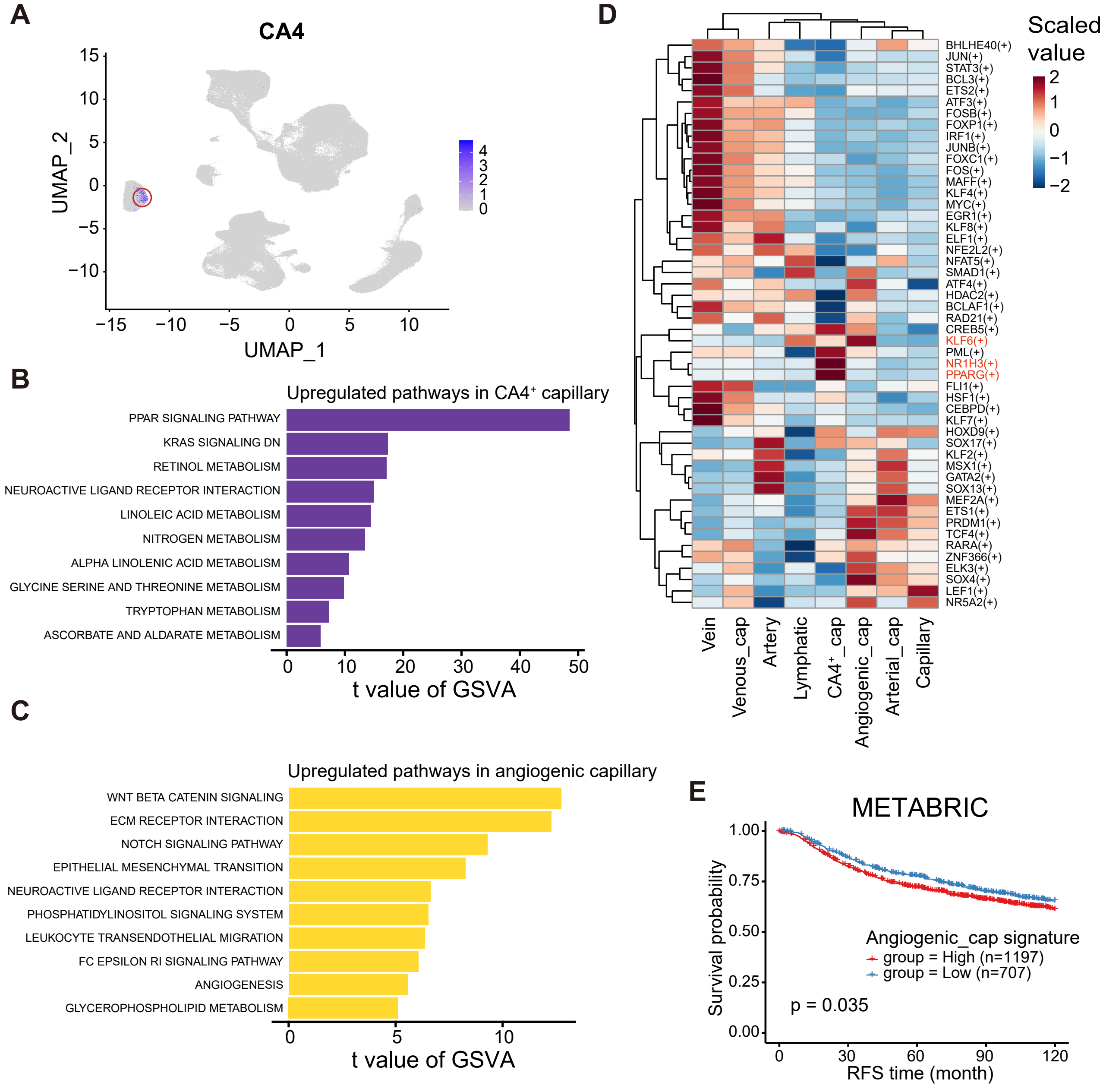
A
CA4
UMAP_2
UMAP_1
15
10
5
0
−5
−10
−15
4
3
2
1
B
Upregulated pathways in CA4⁺ capillary
PPAR SIGNALING PATHWAY
KRAS SIGNALING DN
RETINOL METABOLISM
NEUROACTIVE LIGAND RECEPTOR INTERACTION
LINOLEIC ACID METABOLISM
NITROGEN METABOLISM
ALPHA LINOLENIC ACID METABOLISM
GLYCINE SERINE AND THREONINE METABOLISM
TRYPTOPHAN METABOLISM
ASCORBATE AND ALDARATE METABOLISM
t value of GSVA
20
30
40
50
C
Upregulated pathways in angiogenic capillary
WNT BETA CATENIN SIGNALING
ECM RECEPTOR INTERACTION
NOTCH SIGNALING PATHWAY
EPITHELIAL MESENCHYMAL TRANSITION
NEUROACTIVE LIGAND RECEPTOR INTERACTION
PHOSPHATIDYLINOSITOL SIGNALING SYSTEM
LEUKOCYTE TRANSENDOTHELIAL MIGRATION
FC EPSILON RI SIGNALING PATHWAY
ANGIOGENESIS
GLYCEROPHOSPHOLIPID METABOLISM
t value of GSVA
D
Scaled value
BHLHE40(+)
JUN(+)
STAT3(+)
BCL3(+)
ETS2(+)
ATF3(+)
FOSB(+)
FOXP1(+)
IRF1(+)
JUNB(+)
FOXC1(+)
FOS(+)
MAFF(+)
KLF4(+)
MYC(+)
EGR1(+)
KLF8(+)
ELF1(+)
NFE2L2(+)
NFAT5(+)
SMAD1(+)
ATF4(+)
HDAC2(+)
BCLAF1(+)
RAD21(+)
CREB5(+)
KLF6(+)
PML(+)
NR1H3(+)
PPARG(+)
FLI1(+)
HSF1(+)
CEBPD(+)
KLF7(+)
HOXD9(+)
SOX17(+)
KLF2(+)
MSX1(+)
GATA2(+)
SOX13(+)
MEF2A(+)
ETS1(+)
PRDM1(+)
TCF4(+)
RARA(+)
ZNF366(+)
ELK3(+)
SOX4(+)
LEF1(+)
NR5A2(+)
Vein
Venous_cap
Artery
Lymphatic
CA4⁺_cap
Angiogenic_cap
Arterial_cap
Capillary
E
METABRIC
Survival probability
1.00
0.75
0.50
0.25
0.00
Angiogenic_cap signature
group = High (n=1197)
group = Low (n=707)
p = 0.035
RFS time (month)
60
90
120

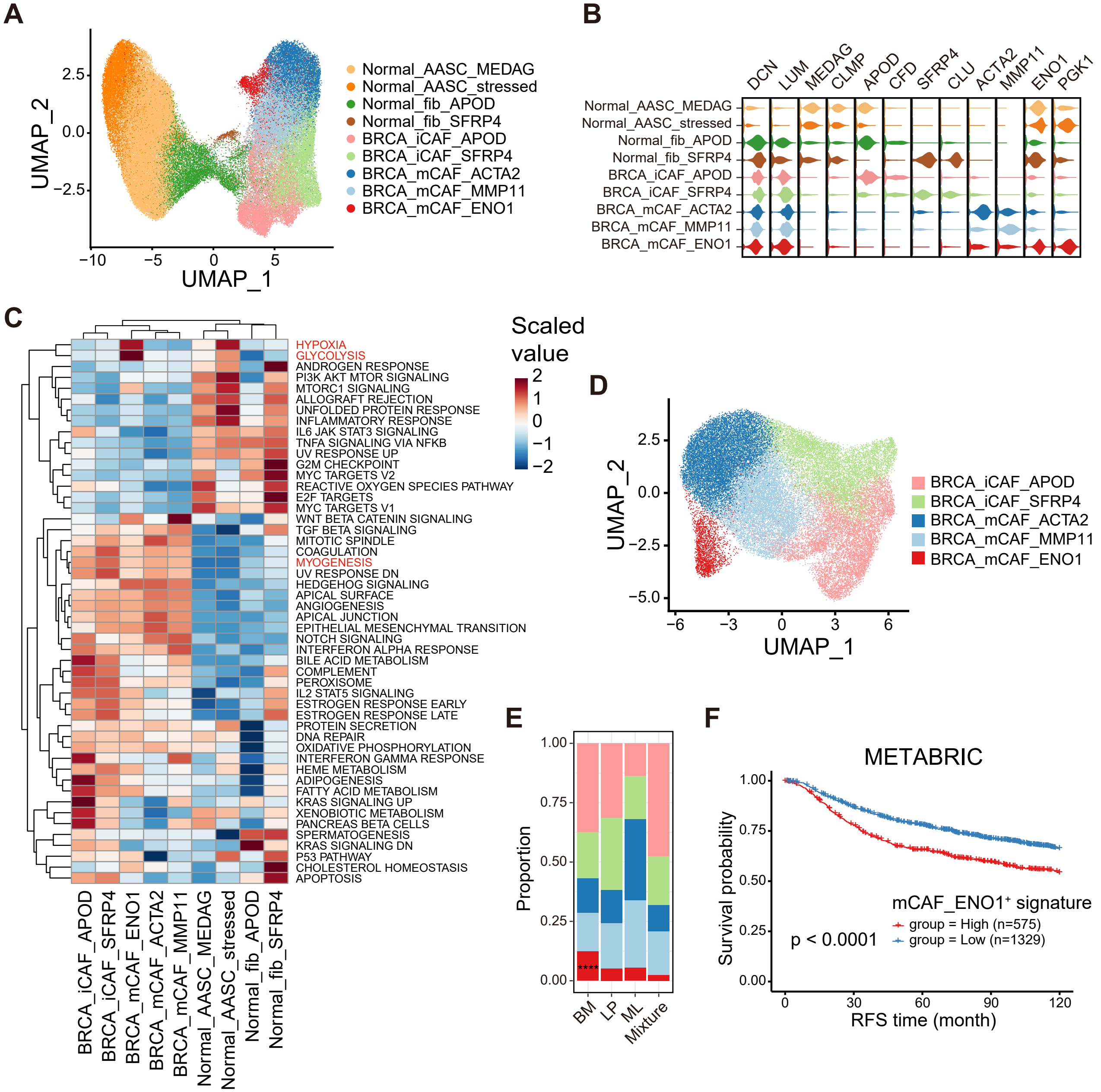

A
UMAP_2
UMAP_1
Normal_AASC_MEDAG
Normal_AASC_stressed
Normal_fib_APOD
Normal_fib_SFRP4
BRCA_iCAF_APOD
BRCA_iCAF_SFRP4
BRCA_mCAF_ACTA2
BRCA_mCAF_MMP11
BRCA_mCAF_ENO1
B
DCN LUM MEDAG CLMP APOD CFD SFRP4 CLU ACTA2 MMP11 ENO1 PGK1
C
Scaled value
HYPOXIA
GLYCOLYSIS
ANDROGEN RESPONSE
PI3K AKT MTOR SIGNALING
MTORC1 SIGNALING
ALLOGRAFT REJECTION
UNFOLDED PROTEIN RESPONSE
INFLAMMATORY RESPONSE
IL6 JAK STAT3 SIGNALING
TNFA SIGNALING VIA NFKB
UV RESPONSE UP
G2M CHECKPOINT
MYC TARGETS V2
REACTIVE OXYGEN SPECIES PATHWAY
E2F TARGETS
MYC TARGETS V1
WNT BETA CATENIN SIGNALING
TGF BETA SIGNALING
MITOTIC SPINDLE
COAGULATION
MYOGENESIS
UV RESPONSE DN
HEDGEHOG SIGNALING
APICAL SURFACE
ANGIOGENESIS
APICAL JUNCTION
EPITHELIAL MESENCHYMAL TRANSITION
NOTCH SIGNALING
INTERFERON ALPHA RESPONSE
BILE ACID METABOLISM
COMPLEMENT
PEROXISOME
IL2 STAT5 SIGNALING
ESTROGEN RESPONSE EARLY
ESTROGEN RESPONSE LATE
PROTEIN SECRETION
DNA REPAIR
OXIDATIVE PHOSPHORYLATION
INTERFERON GAMMA RESPONSE
HEME METABOLISM
ADIPOGENESIS
FATTY ACID METABOLISM
KRAS SIGNALING UP
XENOBIOTIC METABOLISM
PANCREAS BETA CELLS
SPERMATOGENESIS
KRAS SIGNALING DN
P53 PATHWAY
CHOLESTEROL HOMEOSTASIS
APOPTOSIS
D
E
Proportion
BM LP ML Mixture
F
METABRIC
Survival probability
RFS time (month)
mCAF_ENO1⁺ signature
group = High (n=575)
group = Low (n=1329)
p < 0.0001

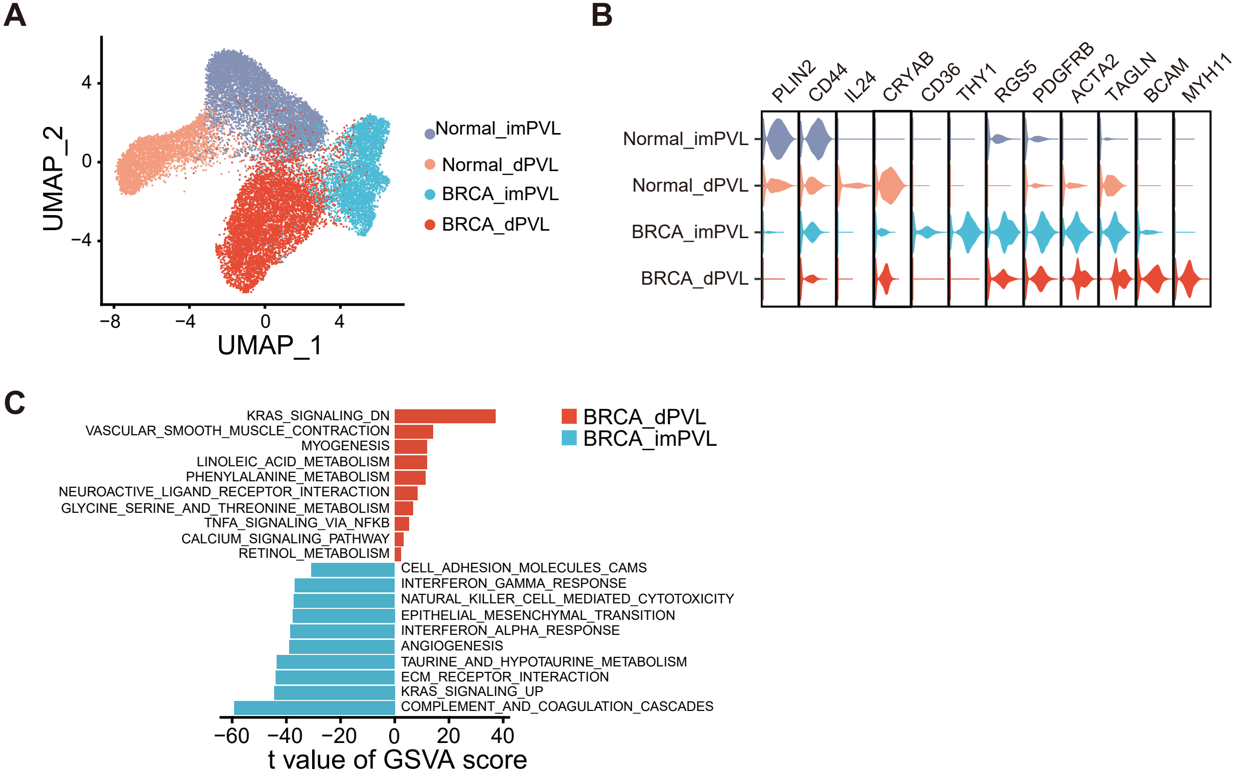
A
UMAP_2
UMAP_1
4
0
−4
−8
−4
0
4
Normal_imPVL
Normal_dPVL
BRCA_imPVL
BRCA_dPVL
B
PLIN2
CD44
IL24
CRYAB
CD36
THY1
RGS5
PDGFRB
ACTA2
TAGLN
BCAM
MYH11
Normal_imPVL
Normal_dPVL
BRCA_imPVL
BRCA_dPVL
C
KRAS_SIGNALING_DN
VASCULAR_SMOOTH_MUSCLE_CONTRACTION
MYOGENESIS
LINOLEIC_ACID_METABOLISM
PHENYLALANINE_METABOLISM
NEUROACTIVE_LIGAND_RECEPTOR_INTERACTION
GLYCINE_SERINE_AND_THREONINE_METABOLISM
TNFA_SIGNALING_VIA_NFKB
CALCIUM_SIGNALING_PATHWAY
RETINOL_METABOLISM
CELL_ADHESION_MOLECULES_CAMS
INTERFERON_GAMMA_RESPONSE
NATURAL_KILLER_CELL_MEDIATED_CYTOTOXICITY
EPITHELIAL_MESENCHYMAL_TRANSITION
INTERFERON_ALPHA_RESPONSE
ANGIOGENESIS
TAURINE_AND_HYPOTAURINE_METABOLISM
ECM_RECEPTOR_INTERACTION
KRAS_SIGNALING_UP
COMPLEMENT_AND_COAGULATION_CASCADES
BRCA_dPVL
BRCA_imPVL
−60
−40
−20
0
20
40
t value of GSVA score
